# Stabilization of mitochondria‐associated endoplasmic reticulum membranes regulates Aβ generation in a three‐dimensional neural model of Alzheimer's disease

**DOI:** 10.1002/alz.14417

**Published:** 2024-12-23

**Authors:** Jacob C. Zellmer, Marina B. Tarantino, Michelle Kim, Selene Lomoio, Masato Maesako, György Hajnóczky, Raja Bhattacharyya

**Affiliations:** ^1^ Genetics and Aging Research Unit MassGeneral Institute for Neurodegenerative Disease Henry and Allison McCance Center for Brain Health Department of Neurology Massachusetts General Hospital, Harvard Medical School Charlestown Massachusetts USA; ^2^ Department of Neuroscience Tufts University School of Medicine Boston Massachusetts USA; ^3^ Alzheimer's Disease Research Unit MassGeneral Institute for Neurodegenerative Disease Massachusetts General Hospital/Harvard Medical School Charlestown Massachusetts USA; ^4^ MitoCare Center Department of Pathology Anatomy & Cell Biology Thomas Jefferson University Philadelphia Pennsylvania USA

**Keywords:** β‐amyloid precursor protein, Alzheimer's disease, amyloid β, endoplasmic reticulum, mitochondria, mitochondria‐associated ER membranes, β‐site APP cleaving enzyme

## Abstract

**INTRODUCTION:**

We previously demonstrated that regulating mitochondria‐associated endoplasmic reticulum (ER) membranes (MAMs) affects axonal Aβ generation in a well‐characterized three‐dimensional (3D) neural Alzheimer's disease (AD) model. MAMs vary in thickness and length, impacting their functions. Here, we examined the effect of MAM thickness on Aβ in our 3D neural model of AD.

**METHODS:**

We employed fluorescence resonance energy transfer (FRET) or fluorescence‐based MAM stabilizers, electron microscopy, Aβ enzyme‐linked immunosorbent assay (ELISA), and live‐cell imaging with kymography to assess how stabilizing MAMs of different gap widths influence Aβ production and MAM axonal mobility.

**RESULTS:**

Stabilizing tight MAMs (∼6 nm gap width) significantly increased Aβ levels, whereas basal (∼25 nm) and loose MAMs (∼40 nm) maintained or reduced Aβ levels, respectively. Tight MAMs reduced mitochondrial axonal velocity compared to basal MAMs, while loose MAMs showed severely reduced axonal distribution.

**DISCUSSION:**

Our findings suggest that stabilizing MAMs of specific gap widths, particularly in axons, without complete destabilization could be an effective therapeutic strategy for AD.

**Highlights:**

- The stabilization of MAMs exacerbates or ameliorates Aβ generation from AD neurons in a MAM gap width‐dependent manner.
- A specific stabilization threshold within the MAM gap width spectrum shifts the amyloidogenic process to non‐amyloidogenic.
- Tight MAMs slow down mitochondrial axonal transport compared to lose MAMs offering a quantitative method for measuring MAM stabilization.

## 1 BACKGROUND

Emerging evidence suggests that specialized mitochondria‐associated endoplasmic reticulum contacts (MERCs), which are biochemically harvested as mitochondria‐associated ER membranes, often referred to as MAMs play a role in several neurodegenerative diseases, including Alzheimer's disease (AD).^1^, ^2^, ^3^ These findings led to the conception of a new hypothesis called the “MAM hypothesis,” which proposes that MAMs, denoted here as MAMs, play a critical role in Aβ generation to initiate the pathogenic cascade of AD, which includes neurofibrillary tangle (NFT) formation, calcium dyshomeostasis, and neuroinflammation.^1^, ^4^ MAMs comprise cholesterol‐rich lipid raft (LR)‐like microdomains in the ER and the outer membrane of mitochondria (OMM) tethered by a series of proteins, creating structural and functional diversity among the MAMs.^5^ MAMs are also implicated in Aβ generation and tau pathology.^6^, ^7^, ^8^ We previously reported that the inhibition of the ER/MAM‐resident sigma‐1 receptor (S1R) downregulated MAM levels, and severely reduced (∼90%) axonal but not somal Aβ generation in a neural model of AD (FAD [familial AD] hNPCs, also called ReN‐GA) in three‐dimensional (3D) dual‐chamber microfluidic devices.^9^, ^10^ S1R is emerging as a unique therapeutic target for mild‐to‐moderate AD. Small molecule S1R‐modulators with favorable safety profiles are gaining attention because they act as anti‐amnestic agents only in pathological conditions but not normal memory.^11^, ^12^, ^13^, ^14^, ^15^, ^16^

The contact thickness or gap widths of MAMs between the ER and mitochondria range between “narrow” (∼6 nm) to “wide” (∼80 nm), while the basal or neutral MAM gap width is ∼25 nm.^17^, ^18^, ^19^, ^20^ Several reports have provided evidence that the length and ER‐mitochondria gap width (thickness) of MAMs contribute to their biological function.^21^ ER stress increased the number of tight MAMs by nearly 2.5‐fold in HeLa cells.^22^ Csordas et al.^20^ reported a significant decrease in the average thickness between mitochondria and the ER from 28.2 to ≅20 nm upon apoptotic stimuli in RBL‐2H3 cells.^20^ How the MAM structure and functions integrate is still unknown. A recent study reported that MAMs formed inside tightly apposed ER and mitochondria (∼10 nm thickness), denoted “full MAMs,” promote apoptosis. In comparison, MAMs formed inside loosely bound ER and mitochondria (∼25 nm thickness), called “medium” MAMs, have antiapoptotic effects.^23^ The tight and loose MAMs also differ in their molecular composition and function.^23^, ^24^ The modulation of these MAMs is an emerging area of research with potential implications for various disorders, including cancer, metabolic disorders, and neurodegenerative diseases.^25^ MAM modulators typically regulate the stability of MAMs by either “loosening” or “tightening” mitochondria–ER contacts.^26^

Using a fluorescence resonance energy transfer (FRET)‐based inducible stabilizer of MAMs in hAPP‐expressing Neuro 2A (N2A_APP_) cells,^27^ we found that tightening MAM gap widths increases Aβ generation in a dose‐dependent manner. Employing RFP‐conjugated constitutive biological linkers to stabilize MAM gap widths of ∼6, 25, or 40 nm (tight, basal, or loose MAMs, respectively) in a 3D human ReN‐cell‐based neural model of AD (ReN‐GA),^28^, ^29^, ^30^ we uncovered new insights into the effect of MAM stabilization on Aβ generation. Our findings indicate that the Aβ levels (basal) in the 3D ReN‐GA model are significantly (*, *p* < 0.05) increased with the stabilization of tight (∼6 nm) MAMs compared to basal (∼25 nm) MAMs. Conversely, stabilizing loose (∼40 nm) MAMs significantly (*, *p* < 0.5) lowers Aβ levels. This shows that the “Aβ‐increasing” tight MAM stabilization is pathogenic, while “Aβ‐lowering” loose MAM stabilization is therapeutic. Additionally, stabilizing tight (∼6 nm) MAMs significantly (*, *p* = 0.0072) increases the axonal accumulation of the MAM‐anchored ER‐mitochondria, denoted here as MitoMERs, and dramatically (***, *p* < 0.0001) reduces their axonal velocity and movement compared to basal (∼25 nm gap width) or ER‐free (> 80 nm gap width) mitochondria. Stabilization of the “Aβ‐lowering” loose (∼40 nm) MAMs severely reduced their axonal distribution.

**RESEARCH IN CONTEXT**

1. **Systematic review**: Emerging evidence suggests that mitochondria‐associated endoplasmic reticulum membranes (MAMs) play a significant role in initiating the amyloid cascade, leading to Alzheimer's disease (AD) pathogenesis. We recently reported that the inhibition of the S1R dramatically reduced Aβ levels, specifically in the axons, by impacting in the MAM stabilization in the neuronal processes or axons. MAM structure and function are diverse. MAM thickness or gap width (tight vs. loose) regulates MAM function. We have found that the stabilization of MAMs formed between tightly (∼6 nm gap width) apposed ER‐mitochondria exacerbates, while those formed between loosely (∼40 nm) ameliorated Aβ levels. MAM modulators typically regulate MAM stabilization by either “loosening” or “tightening” the MAMs. Despite the availability of various MAM modulators, few attempts have been made to test their ability to destabilize MAMs and reduce AD pathology. One reason is that no reliable real‐time quantitative assays exist for measuring MAM stabilization. Moreover, completely disrupting MAMs may be detrimental because MAMs play a critical role in regulating apoptosis. Thus, our goal was to search whether modulating, but not disrupting MAM stability holds therapeutic potential. To address this goal, we used inducible and constitutive biological MAM modulators, as well as pharmaceutical MAM modulating agents to identify that the variation in MAM gap widths play a significant role in exacerbating or ameliorating Aβ generation in pathological neuronal models of AD. During the study, we developed a remarkable quantitative method to evaluate the optimal MAM stabilization needed to cross the threshold from pathogenic (Aβ *increasing*) to nonpathogenic (Aβ *maintaining* or Aβ *lowering*) MAMs.
2. **Interpretation**: Our findings contribute to the growing body of knowledge on the role of MAMs, specifically their gap widths in AD by demonstrating that the stabilization of tightly apposed MAMs (∼6 nm gap width) exacerbates, while the stabilization of loosely apposed MAMs (∼40 nm gap width) ameliorates Aβ generation. This highlights the critical influence of MAM gap width on Aβ pathology and suggests that modulating MAM stability, rather than completely disrupting it, may offer a therapeutic strategy for AD. By developing a novel quantitative method to assess MAM stabilization, our study paves the way for identifying optimal MAM modulators that could prevent AD progression at an early, asymptomatic stage, addressing the lack of reliable real‐time assays for MAM dynamics. These insights could significantly impact future therapeutic approaches aimed at regulating MAM stability to mitigate AD pathology.
3. **Future directions**: We have a very clear future direction. The future steps are as follows:
**a** So far more than 100 proteins have been identified as MAM proteins. These are either MAM‐anchoring or MAM‐resident proteins with various functions. However, all these proteins are not found in every type of MAM. Our immediate next aim will be to determine the protein composition of the axonal and somal MAMs by performing proteomic analysis of these MAMs isolated from the “de‐somatized” axons after axotomy or from bulk neurons. We will extend this study to neurons undergoing MAM modulator treatment.
**b** The functional diversity of MAM structures, specifically their length and gap width, has been identified as a regulator of critical cellular processes, including calcium signaling between the ER and mitochondria. Our immediate next step will be to assess the interplay between local Ca^2+^ dynamics and MAM structures.
**c** Our next goal is to screen a series of known and potential MAM modulators using a three‐dimensional (3D) AD drug screening platform composed of ReN or iPSC‐derived AD models containing neurons or neuron‐microglia co‐culture. We will validate the efficacies of these drugs in a human organoid model of AD developed with our collaborators.
**d** Next, we will carefully select the efficacious MAM modulators with high specificity and efficacy, we will propose to develop clinical trial guidelines to translate our findings to the clinic to treat patients with high risk for AD. For this, we will collaborate with clinicians, regulatory bodies, and relevant stakeholders in the development of clinical trial guidelines.

## 2 METHODS

### 2.1 Experimental models

We used a clonally selected human neural progenitor (ReN)‐derived culture system expressing green fluorescence protein (GFP) and familial AD (FAD) mutations in the amyloid precursor protein (APP) gene (APP^Swe/Lon^) and GFP, both are under the same transcriptional regulation through an IRES element in these cells, termed ReN‐GA. These cells recapitulate AD pathology, namely, Aβ oligomer‐driven NFT when differentiated in a 3D matrix.^28^, ^29^, ^30^ We also used naïve ReN cells (Millipore). ReN cells (naïve and ReN‐GA) were maintained in Dulbecco's modified Eagles medium (DMEM)/F12 media supplemented with L‐glutamine (Gibco), serum‐free B‐27 supplement (Gibco), basic fibroblast growth factor (bFGF), and epidermal growth factor (EGF). For differentiation, the cells were suspended in Matrigel (1:10) to form a 3D matrix and differentiated for 10 days in culture media supplemented with media without bFGF or EGF, as described previously.^9^ Chinese hamster ovary (CHO) and Neuro 2A (N2A) cells were grown in DMEM (Gibco) supplemented with 10% fetal bovine serum (FBS), 10 U/mL penicillin, and 100 µg/mL streptomycin. N2A cells constitutively expressing APP_751_ (N2A_APP_) were used to measure the effect of inducible MAM stabilizers on Aβ generation. Stable cells were maintained in DMEM (Lonza) supplemented with 10% FBS, 100 U/mL penicillin, 100 µg/mL streptomycin, and 2 mM L‐glutamate supplemented with 200 µg/mL G418, as described previously.^27^

### 2.2 Plasmids, reagents, and antibodies

The CFP (cyan fluorescence protein) or YFP (yellow fluorescence protein) ‐linked full‐length ER‐protein phosphatidylinositol‐3‐phosphatase Sac1 phosphatase (FL‐Sac1; ∼70 kD) or mitochondria‐targeting sequence of mammalian A‐kinase anchor protein 1 (AKAP1; 34‐63), denoted as ER‐CFP and Mito‐RFP, respectively, were used as FRET/FLIM biosensors to identify tightly (< 10 nm gap width) formed MAMs. CFP is a donor fluorophore that transfers energy to the acceptor fluorophore YFP when the fluorophores are separated by ≤ 10 nm. To induce tight MAMs we used the expression plasmids encoding the Mito‐YFP or ER‐CFP biosensors fused to the FK506 rapamycin binding (FRB) domain [EMWHEGLEEASRLYFGERNVKGMFEVLEPLHAMMERGPQTLKETSFNQAYG RDLMEAQEWCRKYMK SGNV(K^2095^P)DLTQAWDLYYHVFRRISKQ] or the 12‐kDa FK506‐binding protein (FKBP) domain (MGVEKQVIRPGNGP KPAPG QTVTVHCTGFG KDGDLSQKFW STKDEGQKPF SFQIGKGAVIKGWDE GVIGMQIGEVARLRCSSDYAYGAGGFPAWGIQPNSVLDFEIEVLSVQ), denoted here as FRB‐Mito‐YFP or ER‐CFP‐FKBP, respectively. We generated an expression plasmid by fusing FRB‐YFP‐AKAP1 (34–63) and FKBP‐CFP‐Sac1 (521–587) with a self‐cleaving Tav2A sequence encoding the EGRGSLLTCGDV EENPGP peptide sequence (FRB‐YFP‐AKAP1‐Tav2A‐Sac1‐CFP‐FKBP). The Tav2A‐fused inducible MAM stabilizer, denoted here as MAM‐Tav2A, was generated to obtain equimolar expression levels of Mito‐YFP and ER‐CFP for the ratiometric FRET assay. For constitutive stabilization of MAMs, we used expression plasmids encoding the mitochondria‐targeting sequence of the A‐kinase anchor protein, AKAP1 (34–63), and an ER‐targeting sequence of the ubiquitin‐conjugating enzyme E6, Ubc 6 protein (283–303), linked directly with mRFP (MAM 1X) or containing a 9 or 18 amino acid (aa) linker (MAM 9X or MAM 18X, respectively), as described previously.^23^, ^31^ We used an expression plasmid encoding AKAP1 (34–63) fused with RFP (Mito‐RFP) and a scrambled peptide of 11 aa to mimic the 11 aa of the ER‐targeting Ubc 6 protein (283–303) peptide in the MAM stabilizers. Mito‐RFP was used as a mitochondrial probe.

### 2.3 Purification of desomatized axons

Desomatized pure axons were collected from a cell culture system following published protocol,^32^, ^33^, ^34^ with modification. Briefly, we seed the cells on the upper side of an insert containing a translucent membrane with 0.3 µm diameter pores (Millipore) to allow axons to extend through the pores along the lower membrane surface. After differentiation, the cell bodies on the upper side are removed with a cotton tip applicator, creating desomatized axons on the opposite side. The absence of the nuclear proteins Lamin B1 or NeuN in desomatized axons highlights the purity of the desomatized axons.

### 2.4 Transfection and immunobloting

ReN cells (naïve or ReN‐GA) were electroporated with expression plasmids using a nucleofector kit (Lonza) following the manufacturer's protocol, as described previously.^9^ We used ∼3 × 10^6^ cells and 5 µg of expression plasmid for each electroporation. To transfect CHO_APP_ or N2A_APP_ cells, we used 1 × 10^6^ cells with the appropriate expression plasmids using Effectene (Qiagen) transfection reagent following the manufacturer's protocol.

For Western blot analysis, equal amounts of proteins (∼30 µg) were loaded on NuPAGE 4%–12% Bis‐Tris gels (Invitrogen) for electrophoresis and Western blot analysis as described previously.^35^ Unless otherwise specified, we used 1:1000 and 1:6000 dilutions for the primary and horseradish peroxidase (HRP) ‐conjugated secondary antibodies, respectively, for Western blot assays. The blots were visualized by a LI‐COR Odyssey imaging system. Band intensities were measured by ImageStudio software. Western blots were stripped using stripping buffer (6.25 mM Tris‐HCl, pH 6.8; 0.2% SDS; 0.8% β‐mercaptoethanol) for 30 min at 50°C, followed by thorough washing with phosphate buffered saline (PBS) (5X for 15 min each) before reprobing the blot with different antibodies. Equal amounts of some samples, mostly total cell lysates (TCLs) or total inputs, were occasionally subjected to electrophoresis on separate gels to avoid background signals in the Western blots after two or more stripping processes. The uncropped images of the Western blots are presented in the Supplementary Material. Anti‐mCherry polyclonal antibody (Invitrogen) was used to detect mRFP‐conjugated proteins, anti‐VDAC1 (Abcam) antibody was used as MAM markers, and an anti‐GAPDH (Life Technologies) or anti‐Actin antibodies were used to measure equal loading. For ReN‐GA cells, mGFP levels were also used to measure equal loading.

### 2.5 FACS enrichment of transfected cells

ReN (naïve or GA) or N2A_APP_ cells expressing either the mRFP‐conjugated biomarker (Mito‐RFP) or MAM stabilizers (MAM 1X or MAM 9X) were subjected to fluorescence‐activated cell sorting (FACS) to enrich cells expressing the indicated fluorescent proteins. Briefly, 24 h post‐transfection, the cells were resuspended in PBS supplemented with 2% serum replacement solution (Life Technologies) and 2% B27 and then passed through a cell strainer filter (70 mm Nylon, BD Biosciences). The cell concentrations were adjusted to ∼200,000 cells/mL. Single cells were identified by size (forward scatter laser light) and granularity (90‐degree side scatter laser light). The cells expressing mGFP and mRFP were detected and collected with a BD FACSAria Fusion Cell Sorter (BD Biosciences, San Jose, CA, USA) at the Massachusetts General Hospital (MGH) core facility (Charlestown, MA, USA) as described previously.^36^ The sorted cells were collected in 5 mL or 15 mL tubes with 2/3 mL of DMEM supplemented with 2% FBS or bovine serum albumin (BSA). The sorted/enriched cells were maintained for 48–72 h. The media were exchanged with fresh media 24 h before Aβ release from the FACS‐sorted cells.

### 2.6 Immunostaining and confocal microscopy

Cells differentiated in 2D (N2A_APP_) or 3D Matrigel matrix (ReN or ReN‐GA) were fixed, and the cellular distribution of the fluorescent proteins was analyzed by immunostaining using previously described methods.^9^, ^35^ Briefly, cells were fixed with 3% paraformaldehyde (PFA) for 20 min (for 2D) or 3 h (for 3D) at room temperature. PFA (3%) was made in PBS containing calcium and magnesium. After washing three times with PBS, the cells were blocked using a blocking solution (1% BSA, 0.1% gelatin, 0.1% Triton X‐100, and 0.05% Tween‐20 in PBS containing calcium and magnesium). The cells were then labeled with the appropriate primary antibody solution (1:250) for 1 h or overnight before washing and incubating with the secondary antibody (1:250 dilution) conjugated with the appropriate Alexa Fluor for 45 min. Proteins conjugated with fluorophores, for example, mRFP‐, YFP‐, or CFP‐conjugated MAM stabilizers or mGFP‐conjugated CellLight MitoTracker Green, were imaged directly for confocal microscopy or FRET‐FLIM analysis. As described previously, fluorescence microscopy was performed under a Nikon confocal microscope using 60X or 100X objectives.^9^ The percentage of colocalization of the mRFP‐labeled mitochondrial probe (Mito‐RFP) or constitutive MAM stabilizers (MAM 1X and MAM 9X) with mGFP‐conjugated MitoTracker was determined by performing intensity‐based colocalization after applying color thresholding. Confocal images of the axons were opened with Fiji ImageJ, and the RFP and GFP images were combined and segmented. We applied a color threshold and measured the average fluorescence intensities of the GFP (green) ‐labeled and RFP+GFP merged (yellow) areas. The percentage (%) of colocalization was calculated by dividing the RFP+GFP (yellow) area by the GFP‐labeled area. The analysis was performed on 100 nm segments of axons from approximately five neurons from each set of experiments. We choose each neuron's longest (300–500 m) single neurite as an axon. The distribution of endogenous mitochondria was assessed by labeling differentiated cells with CellLight Mitochondria‐GFP (Invitrogen), which is mGFP‐fused mitochondria‐targeting protein E1 alpha pyruvate dehydrogenase packaged in baculovirus.

### 2.7 Ratiometric spectral FRET and FRET‐FLIM analysis

Spectral FRET and FRET‐FLIM analyses were performed as described previously.^37^, ^38^ Briefly, cells expressing FKBP‐CFP‐ER+FRB‐YFP‐Mito or MAM‐Tav2A were excited using a 440 nm laser. Using a Nikon confocal microscope, fluorescence emitted from the donor FKBP‐CFP‐ER (468–503 nm) and the acceptor FRB‐YFP‐Mito (525–565 nm) were measured. ImageJ was used to measure the average pixel fluorescence intensity after background subtraction, and the emission intensity of YFP over the CFP ratio was used as a readout of the spectral FRET analysis. In FRET‐FLIM, cells expressing FKBP‐CFP‐ER/FRB‐YFP‐Mito or MAM‐Tav2A were excited using a mode‐locked Chameleon Ti: Sapphire laser (Coherent, Inc., Santa Clara, CA, USA) set at 840 nm. The emitted fluorescence was collected using a 60X oil immersion objective through an ET445/58 nm filter (Chroma Technology Corp, Bellows Falls, VT, USA), and the lifetime of the CFP donor fluorophore was measured using a high‐speed photomultiplier tube (MCP R3809; Hamamatsu, Hamamatsu, Japan) and a time‐correlated single‐photon counting acquisition board (SPC‐830; Becker and Hickl GmbH, Berlin, Germany) connected to an Olympus FV3000RS confocal microscope (Tokyo, Japan). SPC image software (Becker and Hickl GmbH) was used to analyze the acquired FLIM data. The donor fluorophore's average lifetime (t2) was measured in the absence of the acceptor fluorophore (for which FRET was absent). In the presence of the acceptor fluorophore, exciting the donor fluorophore results in reduced donor emission energy if the donor and acceptor are ≤ 10 nm apart (FRET present), shortening the lifetime of the donor fluorophore (t2). The t2 was compared to determine the effect of rapamycin treatment on MAM stabilization.

### 2.8 CFP‐ER and Mito‐YFP co‑localization analysis

We performed colocalization analysis as described before.^27^ Briefly, the confocal images of cells expressing MAM‐Tav2A (CFP‐ER and Mito‐YFP) were imported into the Fiji ImageJ. Fluorescent images of single cells were opened and split. The cyan (CFP) and yellow (YFP) channels were converted to eight‐bit images and subjected to Coloc2 plugins analyses to calculate the colocalization parameter, Pearson coefficient (*r*). An “*r*” value of 1 indicates 100% co‐localization, while 0 indicates no co‐localization.

### 2.9 MitoMER counting

To quantify the levels and sizes of the mitochondria‐MAM‐ER (MitoMER) clusters, we opened the ReN‐GA neurons expressing the RFP‐epitope tagged MAM stabilizers MAM 1X or MAM 9X in Fiji ImageJ, setting the scales in micron (µm) unit. We segmented the areas representing the axons and opened both the GFP (ReN‐GA) and the RFP (MAM 1X or 9X) channels, followed by straightening both channels (Edit > Selection > Straighten). Nest, we merged the channels (Image > Color > Merge Channels) arranged (Image > Color > Arrange Channel). We performed “threshold” and “converted to mask,” followed by “watershed” (Process > Binary > Watershed) and, finally, analyzed the numbers and sizes of MitoMER puncta (Analyze → Analyze Particle), setting the limit from 0‐infinity.

### 2.10 Live cell imaging and kymography

Live‐cell imaging and kymography analyses were performed as described previously.^39^ Briefly, cells were plated in 3D Matrigel‐coated glass‐bottom 6‐ or 12‐well plates and differentiated for 10 days. The differentiated neurons were placed in a live cell chamber at 37°C, 5% CO_2_, and 95% humidity. We used a Nikon C2 Eclipse Ti2 inverted confocal microscope to capture fluorescence images using NIS Element AR software at 60X magnification and a resolution of 512 pixels. Supplementary Videos were taken at 1 frame per second for 3 min. To analyze transport, we generated kymographs, and Fiji ImageJ macros were utilized. Vesicles that moved less than 0.1 mm/s were categorized as stationary. The frequency of particle movement was calculated by dividing the number of particles moving in a given direction (anterograde, retrograde) or not moving (stationary) by the total number of particles analyzed in the kymograph. The time each vesicle spent pausing or moving was calculated by averaging the percentage of time spent in each condition for all vesicles in each neuron analyzed. The velocity and run length frequency were calculated considering only moving vesicles for each experimental condition. The analysis was performed on 100 µm axonal tracts for 3 min.

### 2.11 Aβ‐ELISA assays

We measured Aβ (Aβ_40_ and Aβ_42_) levels in 3D differentiated cells, as described previously.^9^ Typically, cells were differentiated for 10 days by changing the media every 2 days. Toxicity was assessed via automated confocal microscopy, which was used to measure changes in mGFP expression, and CytoTox‐ONE Assay (Promega) that quantifies the release of lactate dehydrogenase (LDH) from cells with a damaged membrane. The assay was performed as described previously.^9^ Briefly, fresh conditioned media (CM) was collected every 3 days and mixed with CytoTox‐ONE Reagent in a 96‐well plate. The mixtures were incubated at 22°C for 10 min before adding Cyto‐Tox‐ONE Stop solution. LDH release was measured by recording the fluorescence (Ex = 560 nm and Em = 590 nm). The experiments were performed in triplicate. The percent cell viability was calculated using the experimental data's average fluorescence values, maximum LDH release, and culture medium background for each experiment. We collected the Matrigel fractions by lysing them with 1% Sarkosyl lysis buffer for Aβ measurement. To measure the effect of rapamycin or rapalog on Aβ generation from N2A_APP_ cells expressing MAM‐Tav2A, we collected CM. Aβ (Aβ_40_ and Aβ_42_) species were measured using commercially available electrochemiluminescent ELISA (MSD or WAKO) kits, as described previously.^35^, ^36^ Protein concentrations were determined by a Bio‐Rad BCA assay.

### 2.12 Desomatization

To collect desomatized anucleated axons, we used a cell culture system described by others.^32^, ^33^, ^34^ Briefly, ReN‐GA cells were seeded on the upper side of an insert containing a translucent membrane with 0.3 µm diameter pores (Millipore) to allow axons to extend through the pores along the lower membrane surface. After 10 days of differentiation, the cell bodies on the upper side were scraped with a cotton tip applicator, which created desomatized axons on the opposite side. We purified desomatized axons from differentiated ReN or ReN‐GA cells for biochemical assays or transmission electron microscopy (TEM).

### 2.13 Mouse optic nerve preparation

Optic nerves were isolated from 4‐month‐old nontransgenic wild‐type (WT) mice (male) following the methods described by the Zhigang He laboratory.^40^ Briefly, the heads were separated after perfusion with 2.5% glutaraldehyde and 2.5% PFA in 0.1 M sodium cacodylate buffer (pH 7.4) of fully anesthetized (isoflurane) mice. Next, we removed the skull and removed the brain. The optic nerves rest at the base of the skull. After removing the brains, the optic nerves were dissected by inserting the tips of small scissors into the exposed back of the brainstem immediately before the chiasm to remove the eyes. We moved the eyes with the attached optic nerves into a fresh bath with artificial cerebrospinal fluid (ACSF). We cut the optic nerves as close to the back of the eye as possible. All animal experiments were performed following Institutional Animal Care and Use Committee [IACUC] animal protocol (#2023N000052) and NIH guidelines, including the use of ARRIVE guidelines for reporting animal studies (species, strain, sex, age, source, genetic modifications, housing, and diet).

### 2.14 TEM

TEM analysis was performed according to previous methods.^9^ Briefly, cells or desomatized axons were fixed in a fixative containing 2.5% glutaraldehyde and 2.5% PFA in 0.1 M sodium cacodylate buffer (pH 7.4) overnight at 4°C. After extraction, we cut tissues into small pieces (1–2 mm cubes) for the optic nerves. We immediately fixed them in EM fixative (50 mm sucrose solution, 2.5 mg/mL CaCl_2_, 0.05 M sodium cacodylate buffer, 1.5% PFA and 1.5% glutaraldehyde in water). The cells (or desomatized axons) and tissues were embedded at the Electron Microscopy Core Facility (Harvard Medical School or Massachusetts General Hospital) where these were washed in 0.1 M  cacodylate buffer and postfixed with 1% osmium tetroxide (OsO4)/1.5% potassium ferrocyanide (KFeCN6) for 1 h, washed in water 3x and incubated in 1% aqueous uranyl acetate for 1 h followed by two washes in water and subsequent dehydration in grades of alcohol (10 min each; 50%, 70%, 90%, 2 × 10 min 100%). The samples were then put in propylene oxide for 1 h and infiltrated ON in a 1:1 mixture of propylene oxide and TAAB Epon (Marivac Canada Inc. St. Laurent, Canada). The following day, the samples were embedded in TAAB Epon and polymerized at 60 degrees C for 48 h. Ultrathin sections (approximately 60 nm) were cut on a Reichert Ultra cut‐S microtome, picked up on copper grids stained with lead citrate, and examined with a JEOL 1200EX transmission electron microscope or a TecnaiG^2^ Spirit BioTWIN and images were recorded with an AMT 2k CCD camera.

### 2.15 Statistical analysis

Statistical analyses were performed with GraphPad Prism software. The data are expressed as ± SEMs and are presented as error bars. The number of biological/technical replicates is reported in the figure legends. Curve fitting, nonlinear regression, and statistical analyses were performed using GraphPad Prism. A *p*‐value of 0.05 was used as the significance threshold throughout this study. In all the figures, the *p*‐values are shown as follows: *p* > 0.05 is not significant (ns). * indicates *p* < 0.05, ** indicates *p* ≤ 0.001, and *** indicates *p* ≤ 0.0001. The outliers were identified using the ROUT method.

## 3 RESULTS

### 3.1 Inducing ER‐mitochondria gap widths increased Aβ generation in a dose‐dependent manner

**FIGURE 1:**
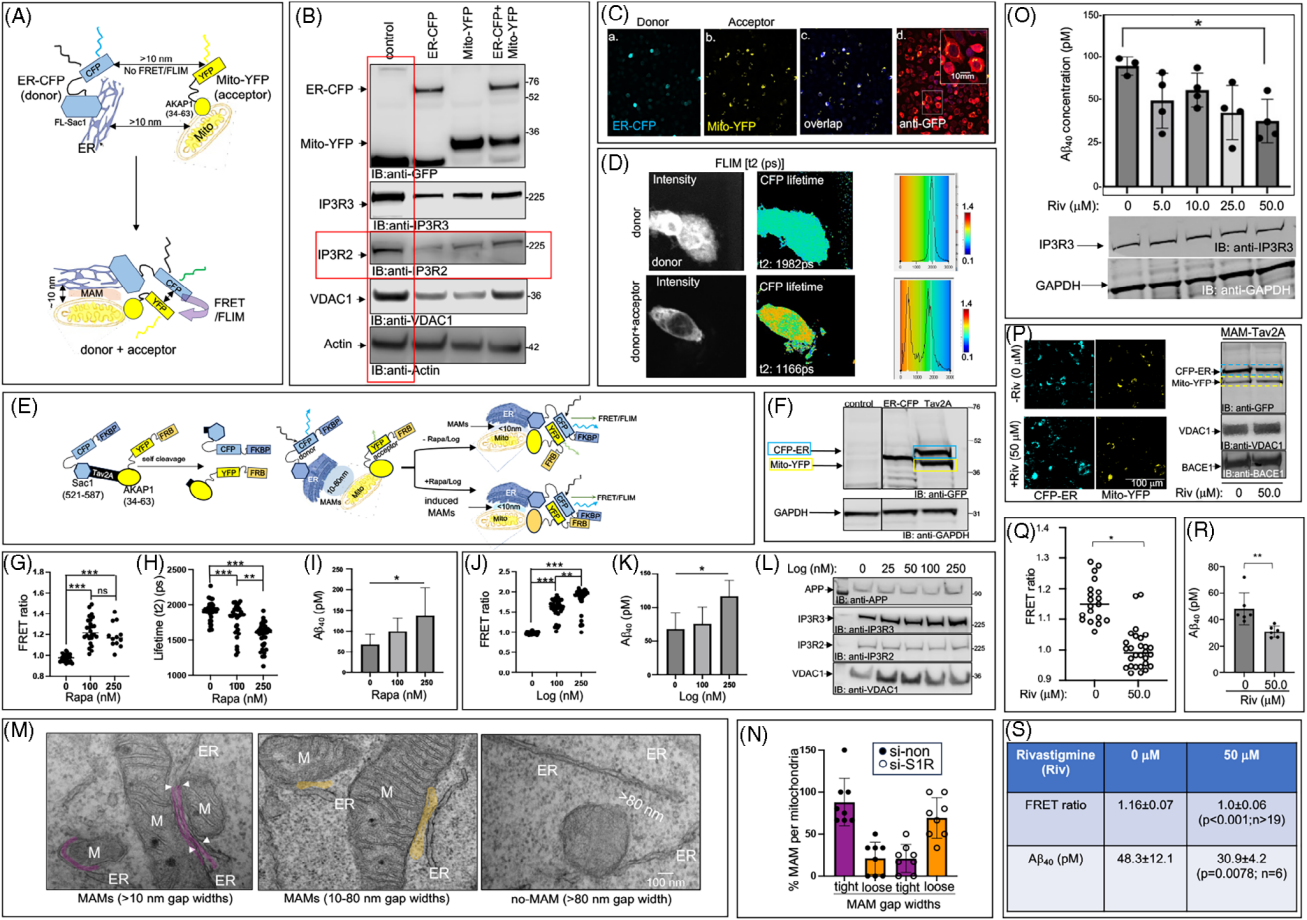
Tightening MAM gap widths increased Aβ generation in a dose‐dependent manner in vitro. (A) Schematic of FRET biosensors ER‐CFP and Mito‐YFP that generates FRET signals from tight MAMs of ≤ 10 nm gap widths. (B) Representative immunoblot of N2A_APP_ expressing the biosensors individually (ER‐CFP or Mito‐YFP) or dually (ER‐CFP + Mito‐YFP). IP3R3 and ACAT1 serve as MAM markers. (C) Direct immunofluorescence (IF) images of cells co‐expressing the donor (ER‐CFP) and the acceptor (Mito‐YFP) (a, b, and c) and indirect IF (iIF) of the cells probed with anti‐GFP antibody that detects both CFP and YFP (d). (D) Representative FLIM analyses measuring the donor lifetime (t2) of cells expressing ER‐CFP (donor) or ER‐CFP+Mito‐YFP (donor+acceptor). (E) Schematic of the inducible MAM stabilizer CFP‐FRB‐ER‐Tav2A‐Mito‐FKBP‐YFP (MAM‐Tav2A) that undergoes self‐cleavage generating equimolar levels of the donor (CFP‐FRB‐ER or ER‐CFP) and the acceptor (Mito‐FKBP‐YFP or Mito‐YFP), and promote FRET/FLIM when attached to the endogenous tight MAMs or upon enhancing the tight MAM formation by inducing the FRB and FKBP interaction with rapamycin (Rapa) or its analog Rapalog (Log). (F) IB of cells expressing MAM‐Tav2A (Tav2A) showing equimolar expression of the CFP‐ER (blue box) and Mito‐YFP (yellow box) after the self‐cleavage of Tav2A. (G, H) FRET (G) and FLIM (H) analysis after rapamycin (Rapa) treatment. FRET values are presented as ratios of intensities between channel 2 (465–500 nm) and channel 1 (525–555 nm). The FLIM (t2) values are presented as donor lifetimes (t2) in pico‐seconds (ps). Forty to 50 cells were randomly selected to generate ROIs on a cell‐by‐cell basis. One‐way ANOVA. *** *p* < 0.0001. (I) Aβ‐ELISA (WACO) assays detecting Aβ_40_ levels in the CM of FACS‐enriched N2A_APP_ cells expressing MAM‐Tav2A after treatment with increasing concentrations of rapamycin (Rapa). One‐way ANOVA. *n* = 3 experiments, sample size ≥ 3. *p ≤ 0.05. Aβ was normalized to the corresponding Rapa or Log treatment by subtracting the value from non‐transfected N2A_APP_ cells. (J, K) FRET ratio (J) and Aβ_40_ levels (K) after rapalog (Log) treatment. (L) Representative Western blot of Log‐treated cells showing little or no impact on the levels of APP and the indicated MAM‐proteins. (M) TEM images showing ER (ER)‐mitochondria (M) contact sites or MAMs of gap widths < 10 nm (purple) or > 10 nm (orange) in ReN‐GA cells electroporated with si‐non or si‐S1R for 48 h. Mitochondria separated by > 80 nm from the ER are non‐MAMs. (N) Quantitation of the tight (< 10 nm) or loose (> 10 nm) MAMs in control (si‐non) and S1R‐silenced (si‐S1R) cells per mitochondria per frame (MAM per M). More than seven frames were used for each analysis. An average of 3–6 mitochondria forming tight or loose MAMs from each frame were manually counted (unbiased, *, *p* < 0.05; **, *p* < 0.001). (O) Aβ ELISA assay of 14‐day 3D‐differentiated ReN‐GA cells after 3‐day rivastigmine (Riv) treatment (once every day). *n* = 4, three independent experiments. One‐way ANOVA. *, *p* < 0.05. (P) Representative confocal (left) and Western blot (right) images of MAM‐Tav2A‐expressing N2A_APP_ cells after 24‐h treatment with Riv (0 or 50 µM). MAM‐Tav2A generated equimolar levels of the donor (CFP‐ER) and acceptor (Mito‐YFP). (Q) Quantitation of the FRET ratio between the donor and the acceptor of vehicle‐ (0 µM) or Riv‐treated (50 µM) MAM‐Tav2A‐expressing N2A_APP_ cells. (R) Aβ ELISA (Wako) of CM of vehicle‐treated (0 µM) or Riv‐treated (50 µM) cells showed a significant reduction of Aβ_40_ (pM) levels after 50 µM Riv‐treatment. *n* = 6, *p* < 0.001. (S) FRET (ratio) and Aβ (pM) values are tabulated. APP, amyloid precursor protein; CFP, cyan fluorescence protein; CM, conditioned media; ELISA, enzyme‐linked immunosorbent assay; ER, endoplasmic reticulum; FKBP, FK506‐binding protein; FRB, FK506 rapamycin binding; FRET, Fluorescence resonance energy transfer; GFP, green fluorescence protein; MAM, mitochondria‐associated ER membrane; TEM, transmission electron microscopy; YFP, yellow fluorescence protein.

MAM‐stabilizing S1R is emerging as a unique therapeutic target for AD, with small molecule modulators exhibiting favorable safety profiles and anti‐amnestic effects in mild‐to‐moderate AD.^11^, ^12^, ^13^, ^14^, ^15^, ^16^ S1R are highly expressed in the brain and localized in the ER or MAMs. S1R modulators are gaining attention because they act as anti‐amnestic agents only in pathological conditions but not in normal memory. We previously analyzed the TEM images of ReN‐GA cells and reported that the number of ER‐mitochondria contacts containing no visible electron‐dense areas (considered as MAMs) dramatically reduced after knocking down S1R (S1R‐KD) using a SMART‐pool of siRNA against S1R (si‐S1R).^9^ We also demonstrated that the inhibition of S1R caused the downregulation of MAMs inside axons and dramatically decreased (∼90%) axonal Aβ generation without altering somal Aβ generation, suggesting that axonal MAMs differ from somal MAMs and are needed for axonal Aβ. The differential distribution of the MAMs in the soma and axons is not surprising because several reports have provided compelling evidence that MAMs differ structurally and functionally due to their diverse sizes and cellular distribution. For example, MAM gap width (vertical) and length (horizontal) determine their size, cellular distribution, and biological functions (reviewed in ref. [^21^]). The ER structures determine MAM gap widths. The ribosome‐rich rough ER (RER), found primarily in the somatodendritic area forms tighter MAMs compared to MAMs formed by the tubular or smooth ER (SER) found predominantly in the axons and synapses. MAM thickness or gap width ranges between tight (∼6 nm) to loose (∼80 nm), while the basal or neutral MAM widths are ∼25 nm ^18^, ^19^, ^20^. Live FRET imaging of neurons from AD transgenic rats revealed significant perturbation of tight MAMs (< 10 nm) but no alteration of loose MAMs (∼20 nm) compared to MAMs from the WT.^41^ Thus, to fully understand the effect of S1R knockdown (S1R‐KD) on MAMs of various gap widths, we employed the ER‐ and mitochondria‐targeting biosensors, ER‐CFP and Mito‐YFP (Figure 1A), respectively. These were designed to detect and quantify the levels of tight (< 10 nm gap width) MAMs versus loose or non‐MAMs. The donor (CFP) and the acceptor (YFP) fluorophores generate a FRET signal when ≤ 10 nm apart, making them ideal for quantifying the degree of tight (6–10 nm) MAMs. We expressed ER‐CFP and Mito‐YFP in N2A_APP_ cells (Figure 1B and C). We evaluated the FRET by measuring the lifetime (t2) of the donor fluorophore in the absence or presence of the acceptor fluorophore in N2A_APP_ cells (Figure 1D). The lifetime of the donor decreased in the presence of the acceptor (Figure 1D, donor [t2 = 1982 ps,] and donor+acceptor [t2 = 1166 ps]), suggesting that a t2 value of 1166 ps may be used as a measure of tight MAMs, while a t2 value of 1982 can be used as a measure of loose or non‐MAMs (> 10 nm gap widths).[Fig alz14417-fig-0002]

To examine whether the tightening of MAMs directly impacts Aβ generation, we measured Aβ levels from N2A_APP_ cells after inducing tight MAM formation in a dose‐dependent manner. In this system, we used expression plasmids encoding the FKBP‐fused Mito‐YFP and or FRB‐fused ER‐CFP. The FKBP‐CFP‐ER and Mito‐YFP‐FRB were designed to “tighten” MAM gap widths upon the addition of rapamycin in a dose‐dependent manner, and increase FRET signal.^20^ To fully exploit rapamycin‐inducible FKBP‐FRB dimerization to quantitatively measure the impact of increasing MAM “tightening” on Aβ generation, we introduced a self‐cleavable viral Tav2A sequence ^42^ between the two encoding FRB‐YFP‐Mito and FKBP‐CFP‐ER. The inducible MAM stabilizer is denoted here as MAM‐Tav2A (Figure 1E). Probing the TCLs of N2A_APP_ cells expressing MAM‐Tav2A with an anti‐GFP antibody, we detected equimolar expression levels of the donor ER‐CFP (predicted mol. wt. ∼49 kDa) and acceptor Mito‐YFP (predicted mol. wt. ∼44 kDa) (Figure 1F, blue and yellow boxes, respectively). Ratiometric FRET and FLIM analyses of the MAM‐Tav2A‐expressing FACS‐enriched N2A_APP_ cells (Figure S1) cells revealed a dose‐dependent increase in the FRET ratio and a decrease in donor lifetime (t2) when treated with rapamycin (Rapa) (0, 100, and 250 nM) (Figure 1G,H, respectively). We also detected does‐dependent increase in Aβ_40_ levels upon Rapa treatment (Figure 1I). Rapamycin inhibits the mammalian target of the rapamycin (mTOR) signaling pathway to downregulate TREM2 (Triggering receptor expressed on myeloid cells 2) in microglia and reduce Aβ plaque clearance in AD mice (5XFAD),^43^ we also tested the effect of the rapamycin analog AP21967, known as rapalog (Log), which binds poorly to mTOR and thus is relatively nontoxic to cells but can bind to the FRB domain with equal affinity as rapamycin due to a compensatory cavity‐forming mutation, K^2095^P, introduced in the FRB domain.^44^ Rapalog treatment also increased the FRET signals and Aβ_40_ generation in a dose‐dependent manner (Figure 1J,K, respectively). Rapalog treatment exhibited little or no effect on the levels of APP or other MAM proteins, such as IP3R3 or VDAC1 (Figure 1L). Given that VDAC1 can bind other IP3Rs, such as IP3R1/2, we also evaluated the effect of rapalog on IP3R2 levels, which remained unaltered (Figure 1L).

To further confirm the direct correlation between MAM “tightening” and Aβ generation, we revisited the TEM images of S1R‐KD ReN‐GA cells and counted the tightly apposed ER and mitochondria (with no visible electron‐dense area) as tight MAMs (< 10 nm) (Figure 1M, purple) and the ER and mitochondria separated by visible electron‐dense areas ranging between 10 and 80 nm as loose MAMs (Figure 1M, orange). Mitochondria and ER separated by > 80 nm were not considered as MAMs (Figure 1M, no‐MAMs). Quantitation revealed a dramatic reduction in the number of tight MAMs (< 10 nm gap width) in si‐S1R‐containing cells compared to cells containing scrambled siRNA control (si‐non), as expected.^9^ However, the number of loose MAMs (10‐80 nm gap widths) was increased by ∼2.5‐fold (**, *p* < 0.001) (Figure 1N).

Many pharmacological agents disrupt MAMs by “tightening” or “loosening” the ER‐mitochondria contacts and are currently under preclinical and clinical trials for many diseases, including cancer and metabolic disorders.^25^ Among these, the S1R modulators exhibit diverse chemical structures and safe therapeutic/pharmacological profiles showing promise in improving memory among dementia patients.^45^, ^46^ Notably, the anti‐amnestic S1R ligand rivastigmine is a United States Food and Drug Administration (FDA) ‐approved drug, currently under preclinical studies for mild or moderate AD.^47^, ^48^ Rivastigmine modulates MAM stabilization by regulating the levels of MAM‐anchoring mitofusin‐2 (MFN2).^49^ As a proof‐of‐concept study to determine whether the modulation of MAM “tightness” lowered amyloid pathology, we examined the effect of rivastigmine on Aβ generation from ∼12‐day differentiated ReN‐GA cells after 3‐day treatment (once every day). We measured the Aβ release in the CM by performing an Aβ‐ELISA (Wako) assay. We detected a dose‐dependent reduction of the neurotoxic Aβ_42_ levels, reaching significance at 50 µM rivastigmine (Figure 1O). Our result is consistent with several studies demonstrating that rivastigmine treatment lowers Aβ levels in cultured neurons and AD mice (3XTg).^50^, ^51^

To examine whether rivastigmine treatment modulated MAM tethering, we evaluated the effect of 50 µM rivastigmine in N2A_APP_ cells expressing Tav2A (Figure 1P). The equimolar expression levels of the CFP‐ER and Mito‐YFP remained unaffected upon rivastigmine (50 µM) treatment. Rivastigmine also had no effect on the expression levels of VDAC1 or BACE1 (Figure 1P, IB: anti‐VDAC1 or anti‐BACE1). In contrast, the rivastigmine (50 µM) treatment significantly (*p* < 0.001) reduced the FRET signals (Figure 1Q), indicating MAM loosening. The rivastigmine treatment, on the other hand, did not affect the colocalization frequencies of the CFP‐ER and Mito‐YFP (Figure S2). Together, the results suggested that the rivastigmine “loosened” the MAM gap widths without disrupting the ER and mitochondria apposition. However, Aβ_40_ levels were significantly reduced upon rivastigmine (50 µM) treatment (Figure 1R). Quantitation revealed that rivastigmine (50 µM) lowered the FRET ratio from 1.16 ± 0.07 (0 µM) to 1.0 ± 0.06 (50 µM), and reduced the levels of Aβ_40_ from 48.3 ± 12.1 to 30.9 ± 4.2 pM (Figure 1S).

Together, our results provide the first proof‐of‐concept that MAM gap width “tightening” increases while “loosening” decreases Aβ generation in the pathological condition.

### 3.2 Validation of the effectiveness of biological linkers in stabilizing the MAMs of fixed gap widths in ReN‐GA neurons

**FIGURE 2 alz14417-fig-0002:**
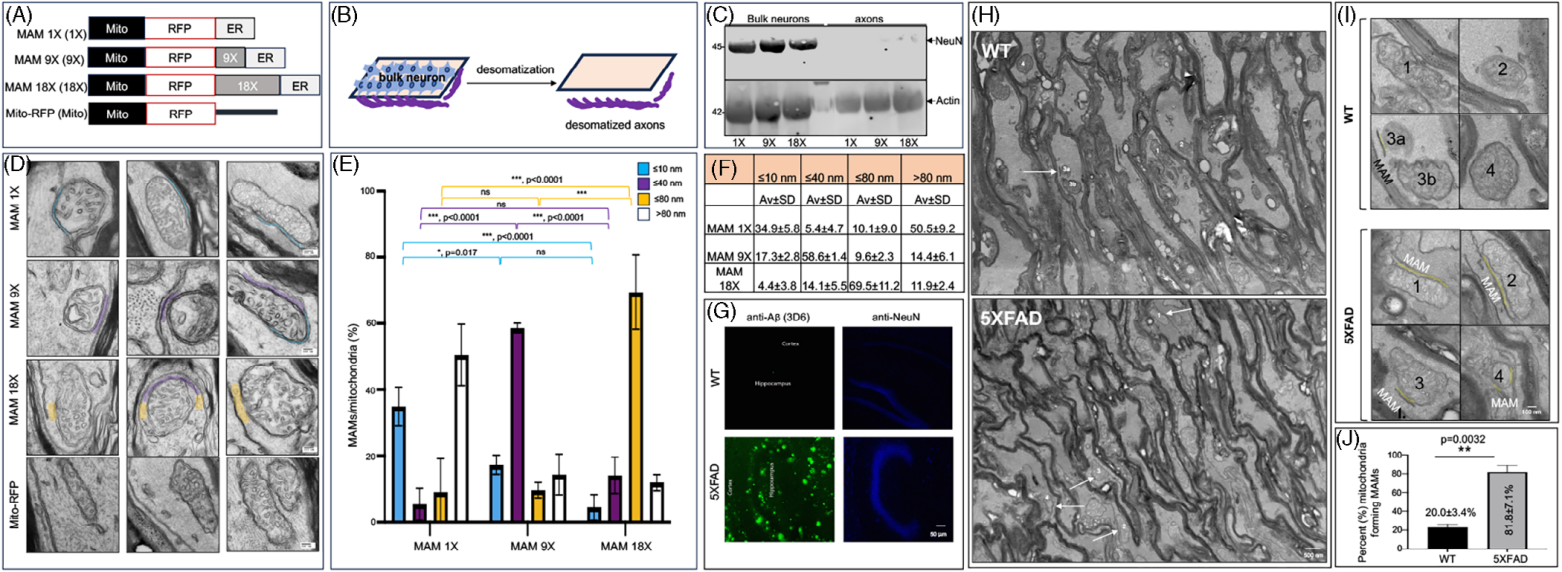
The constitutive MAM stabilizers MAM 1X, MAM 9X, and MAM 18X increased the amounts of tight, basal, and loose MAMs in the axons. (A) Schematics of the constitutive MAM stabilizers MAM 1X (1X), MAM 9X (9X), and MAM 18X (18X) generated by fusing the mitochondrial targeting sequence of AKAP1 (34–63) (Mito) with the ER‐targeting sequence of Ubc6 (283–303) (ER) with RFP. Mito‐RFP (Mito) represents the Mito conjugated with RFP. Mito‐RFP contains a scrambled peptide (black line) (DLELKLRILQSTVPRARDPPVAT) to mimic the length of Ubc6 (283‐303) in the MAM stabilizers. (B) Schematic of the desomatization process of ReN‐GA cells seeded on membrane inserts with 0.3 µm^2^ pores and differentiated for 7–12 days before isolating the bulk neurons and desomatizing the anucleated axons. Bulk neurons were isolated by scraping the cells from the upper side of the insert. To obtain pure axons, the bulk neurons on the upper side were scraped with a cotton tip applicator, which created desomatized axons on the opposite side. (C) Western blot of protein extracts from bulk neurons and purified anucleated desomatized axons from 10–14 days differentiated ReN‐GA cells probed with antibodies against the nuclear protein NeuN (anti‐NeuN) or with anti‐Actin antibody. NeuN was largely absent in the desomatized axons, confirming the isolation of pure desomatized anucleated axons. (D) Representative EM images (triplicates) of axons from MAM 1X, MAM 9X, MAM 18X, or Mito‐RFP expressing 12‐day differentiated ReN‐GA neurons. MAMs of ≤ 10 nm (blue), ≤ 40 nm (purple), or ≤ 80 nm (orange) were identified (manually, unbiased) per mitochondria. (E) Quantitative analysis of the number of MAMs of gap widths ≤ 10 nm, ≤ 40 nm, or ≤ 80 nm or non‐MAMs (> 80 nm gap width) per mitochondria per frame. More than four frames were used for each analysis. An average of 3–6 mitochondria forming or not forming MAMs from each frame was manually counted (unbiased, *, *p* < 0.05; ***, *p* < 0.0001). (F) Table recording the average (Av) number of ≤ 10 nm, ≤ 40 nm, ≤ 80 nm or > 80 nm gap widths per mitochondria per frame. (G) Representative confocal images of the WT and 5XFAD mouse brain sections immunostained with anti‐Aβ antibody (3D6) and anti‐NeuN. (H) Representative 5000X magnified TEM images of the WT and 5XFAD mouse optic nerve axons. Arrows indicate mitochondria forming MAMs. (I) Four representative 25000X magnified images from the 5000X magnified images of each genotype (WT and 5XFAD). The 25000X magnified images were used to assess the number of MAMs (yellow areas juxtaposed between the ER and mitochondria). (J) Quantitation of the percentage of mitochondria forming MAMs inside the optic nerve axons of the WT and 5XFAD mice. More than four frames were used for each analysis. Total and MAM‐forming mitochondria from each image were manually counted (unbiased, ** *p* = 0.0032). EM, electron microscopy; ER, endoplasmic reticulum; FAD, familial Alzheimer's disease; MAM, mitochondria‐associated ER membrane; SD, standard deviation; TEM, transmission electron microscopy; WT, wild‐type.

MAMs are transient structures because of the highly dynamic nature of mitochondria ^52^ and the ER.^53^, ^54^ To investigate the effect of MAM gap widths on Aβ in pathological conditions, we introduced two well‐characterized constitutive MAM stabilizers in our 3D ReN‐GA model of AD. These are expression plasmids encoding mitochondria targeting sequence of AKAP1 (34–63) and ER‐targeting sequence of the Ubc 6 protein (283–303) linked directly with RFP (MAM 1X) or containing a 9‐ or 18 (repeat of the 9)‐amino acid linkers (MAM 9X or MAM 18X, respectively) (Figure 2A). MAM 1X stabilizes MAMs of gap width ∼6 nm (tight MAMs). In contrast, MAM 9X and MAM 18X stabilize MAMs of ∼24 and 40 nm gap widths, respectively.^20^, ^23^, ^31^ We used an RFP‐conjugated AKAP1 (34–63) (Mito‐RFP) as a control (Figure 2A). To validate the effectiveness of these linkers in their ability to stabilize the MAMs of fixed gap widths (∼6, 24, or 40 nm) in ReN‐GA cells, we conducted a comprehensive quantitative analysis using TEM images of FACS‐enriched ReN‐GA cells overexpressing MAM 1X, MAM 9X, or MAM 18X (Figure 2B). We performed the TEM image analysis specifically on the desomatized axons rather than on bulk neurons, with the primary objective of investigating the role of axonal MAM gap widths in amyloid pathology. For this, we collected pure axons via desoamatization of neurons following a previously published protocol.^32^, ^33^, ^34^ We seeded the FACS‐enriched ReN‐GA cells expressing MAM 1X, MAM 9X, or MAM 18X on the upper side of an insert containing a translucent membrane with 0.3 µm diameter pores (Millipore) to allow axons to extend through the pores along the lower membrane surface. After differentiation, the neurons (bulk neurons) on the upper side were removed with a cotton tip applicator, creating desomatized axons on the opposite side (Figure 2B). We confirmed that the nuclear protein NeuN (anti‐NeuN) was largely absent in desomatized axons, highlighting pure axons (Figure 2C). We prepared the purified desomatized axons and performed TEM imaging (Figure 2D), as described in Figure 1. Our quantitative analysis revealed that 34.9 ± 5.8% of mitochondria formed tight (≤10 nm) MAMs in the axons of MAM 1X‐expressing cells, compared to 17.3 ± 2.8% and 4.4 ± 3.5% in MAM 9X‐ or MAM 18X‐expressing cells, respectively (Figure 2D,E). Conversely, a significantly higher number of MAMs with gap widths ≤40 nm or ≤80 nm was detected in the axons of MAM 9X‐ or MAM 18X‐expressing cells, respectively (*p* < 0.0001, *n* ≈ 40, 3 separate TEM analyses) (Figure 2F). These findings are consistent with previously described results in RBL‐2H3 mast cells,^31^ validating the biological linkers as effective stabilizers of tight, basal, and loose MAMs in ReN‐GA axons. We also performed a confocal imaging analysis of naïve ReN cells expressing the RFP‐labeled MAM stabilizers after labeling with GFP‐conjugated mitotracker (Invitrogen) to find strong colocalization of each stabilizer with the mitotracker and no cytoplasmic or axoplasmic distribution confirming no mislocalization of the stabilizers (Figure S3). The desomatization method allowed us to isolate pure axons but not pure soma. However, we still quantified MAMs in the soma by analyzing TEM images showing visible nuclei or Golgi apparatus, which are typically absent in axons (Figure S4). This analysis revealed no significant difference in the frequencies of tight versus loose MAMs in the soma (data not shown). To gain a better understanding, more comprehensive TEM analysis of entire neurons is needed, and modified dual‐chamber microfluidic devices, previously used to measure axonal and somal Aβ levels,^10^, ^11^ may help address these challenges and provide insight into MAM distribution and their role in Aβ generation.

Collectively, our results suggested that stabilization of MAMs of different gap widths produced different effects on Aβ levels in AD neural model. Employing a 2D–3D dual chamber microfluidic system that could separate axons or axonal micro‐environments from bulk neurons or somal environments, we also reported before that S1R‐antagonsist NE‐100 downregulated MAM levels and severely reduced axonal Aβ, whereas the S1R‐agonist PRE‐084 increased axonal Aβ levels from ReN‐GA neurons. We must highlight that the axons of ReN‐GA neurons do not form protective myelin sheaths in the absence of oligodendrocytes. Thus, the axonal Aβ accumulating in the axonal chambers was likely the pool of Aβ formed inside axons or transported from the somatodendritic compartment being released into the axonal chambers. Notably, the extraneuronal Aβ is secondary to the generation of intraneuronal Aβ.^55^

Aβ accumulation in the retina precedes Aβ deposition in AD mouse models.^56^, ^57^ Mouse optic nerves serve as a reliable model of pure axons because 85‐90% of axons arising from the nasal retina project through the contralateral optic tract to the contralateral brain.^58^, ^59^ The optic nerve consists exclusively of retinal ganglion cell (RGC) axons and the glia and vasculature, which support the RGC axons (Figure S5). In tandem with the ability to easily manipulate RGCs, this allows for easy quantification of experimental changes within the axons. Thus, to assess the significance of axonal MAMs in AD, we isolated optic nerves from 6‐month‐old WT B6/SJL and transgenic 5XFAD mice. The 5XFAD exhibited significant amyloid plaque accumulation in brain sections labeled with the anti‐Aβ (3D6) antibody, as expected (Figure 2G). TEM analysis of the optic nerves (Figure 2H,I) revealed an approximately four‐fold increase in the percentage of mitochondria forming MAMs within optic nerve axons in 5XFAD mice compared to WT (81.8 ± 7.1% in 5XFAD vs. 20 ± 3.4% in WT; *p* = 0.0032; *n* ≥ 3 images from three mice per group) (Figure 2J). Here, we have only used male mice. Female 5XFAD mice develop amyloid pathology earlier than males, which could introduce variability and complicate the interpretation of results, so using male mice might help ensure more consistent data. Our findings establish a link between elevated axonal MAMs and AD in vivo. However, further research is needed to determine whether the substantial increase in MAMs in AD axons is a cause or consequence of amyloid pathology.

### 3.3 Stabilization of the tight MAMs exacerbated, while the loose MAMs ameliorated Aβ generation from 3D ReN‐GA neurons

The implications of the structural characteristics of the ER for MAM functions are not fully understood, possibly because the different conformations of the ER (rough vs. smooth) are not distinctly separated in most cells. However, neurons bypass this complication due to their intriguing cellular architecture characterized by pronounced polarization. The distinct separation of axons, predominantly housing SERs, from soma containing both SERs, RERs, and other forms of ER, such as cortical ER,^60^ provides an unprecedented opportunity to study the role of tight versus loose MAMs, which may have profound implications for mitochondrial integrity and transport in the development of neurodegenerative diseases.

**FIGURE 3:**
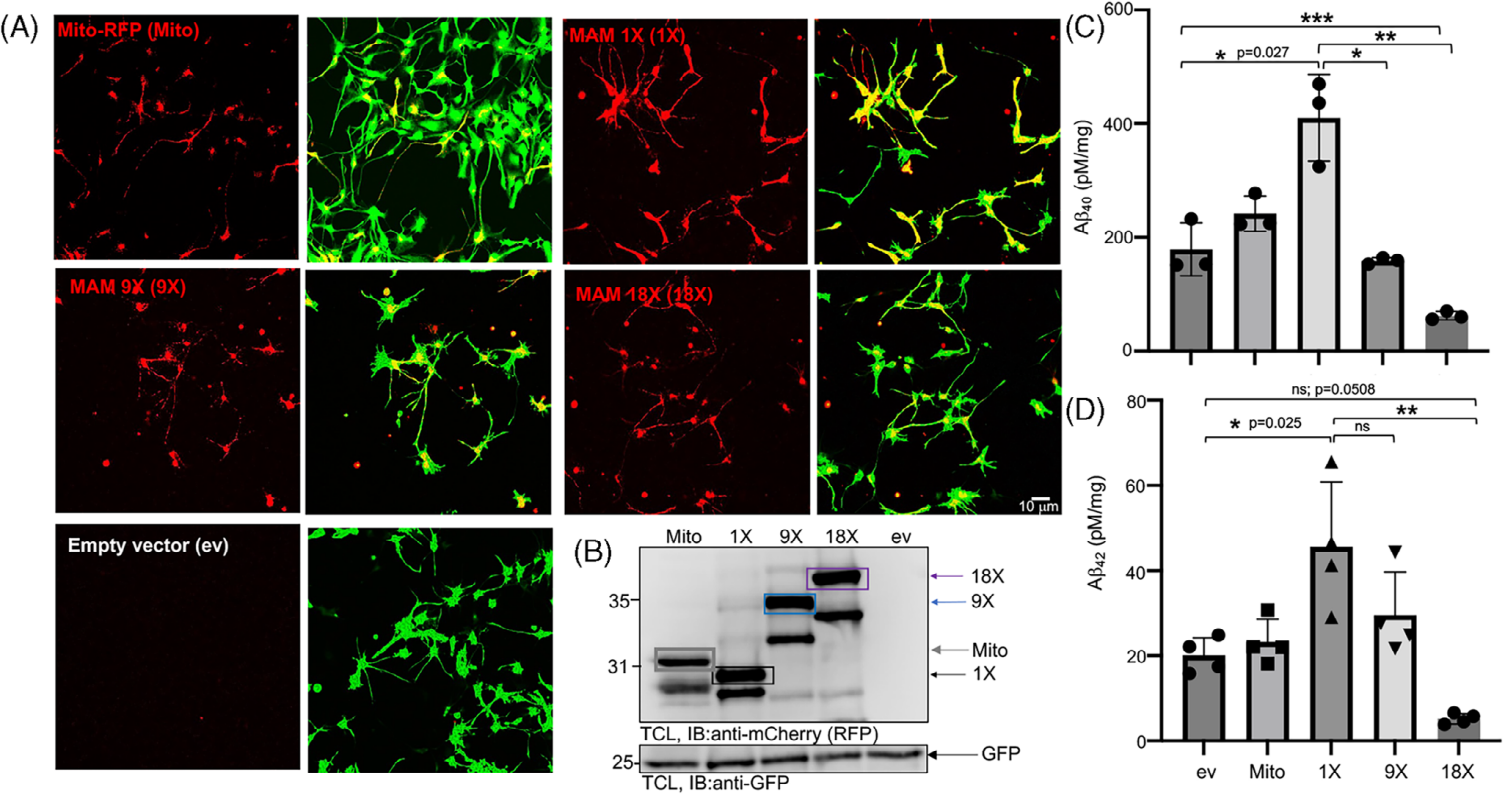
MAM stabilization increased Aβ generation in a 3D neural cell culture model of AD. (A) Representative confocal images of 12‐day 3D differentiated ReN‐GA cells expressing Mito‐RFP, MAM 1X, MAM 9X, MAM 18X, or ev. Punctate labeling of Mito‐RFP, MAM 1X, and MAM 9X was detected in the soma and neuronal processes or axons (red). (B) Representative immunoblot image of the expression levels of Mito‐RFP (gray box), MAM 1X (black box), MAM 9X (blue box), or MAM 18X (purple box). (C, D) SDS‐soluble Aβ_40_ (C) and Aβ_42_ (D) were collected from the 3D matrix and measured via MSD ELISA. One‐way ANOVA was performed; *n* = 3 experiments (sample size ≥ 3). *, *p* < 0.05. The significance of the differences was evaluated against untransfected (control) ReN‐GA cells. AD, Alzheimer's disease; ELISA, enzyme‐linked immunosorbent assay; ev, empty vector; MAM, mitochondria‐associated ER membrane.

We enriched the ReN‐GA cells expressing Mito‐RFP, MAM 1X, MAM 9X, or MAM 18X using FACS as before.^9^ An equal number (30,000 per well) of FACS‐enriched cells were plated in a 3D Matrigel (10%) matrix. Both confocal microscopy and Western blot analysis detected stable and equal expression levels of Mito‐RFP, MAM 1X, MAM 9X, and MAM 18X up to 12 days of differentiation (Figure 3A,B, respectively). A decrease in the expression levels of the stabilizers was observed after 14–15 days of differentiation (*data not shown*), prompting us to center our studies around cells differentiated for 12 days. The viability of the cells was assessed by LDH analysis of the CM. Electrochemiluminescent MSD (MesoScale Discovery) of 1% Sarkosyl extract detected approximately two‐fold increased Aβ (Aβ_40_ and Aβ_42_) levels in MAM 1X‐expressing neurons compared to the empty vector (ev)‐transfected (Figure 3C,D, 1X vs. ev). Mito‐RFP or MAM 9X expression, on the other hand, exhibited no significant change in Aβ levels compared to the control ev (Figure 3C,D, Mito or 9X, respectively). Most importantly, cells expressing MAM 18X resulted in a significant (**, *p* < 0.001) loss of Aβ (Aβ_40_ and Aβ_42_). Our studies revealed that stabilizing MAMs characterized by their tightest profile (∼6 nm gap width) promoted Aβ generation. In comparison, stabilizing loose (∼40 nm) MAMs, representing the weaker end of the MAM gap width spectrum, reduced Aβ generation. These findings indicate that stabilizing MAMs of varied gap widths impacts Aβ generation in pathological conditions.

Our earlier report demonstrated that the modulation of MAM levels or stabilization regulated the axonal Aβ levels, which was accumulated inside the axonal chambers of a 3D dual chamber microfluidic system that could separate axonal micro‐environments from somal environments. Axonal Aβ was likely the pool of Aβ formed inside axons or transported from the somatodendritic compartment being released into the axonal chambers of ReN‐GA neurons that do not form protective myelin sheaths in the absence of oligodendrocytes. Extraneuronal Aβ is secondary to the generation of intraneuronal Aβ.^55^ Thus, our current result suggesting that the stabilization of MAMs of increasing gap widths would restore or lower the levels of Aβ is consistent with our current earlier report. Here, we conclude that the stabilization of the basal or loose MAMs would serve as nonpathogenic MAMs by “*maintaining*” or *“lowering*” as opposed to “*increasing”* Aβ levels.

### 3.4 Constitutive stabilization of the ER‐mitochondria connections via loose MAMs resulted in a severely reduced axonal distribution compared to tight or basal MAM stabilization

**FIGURE 4:**
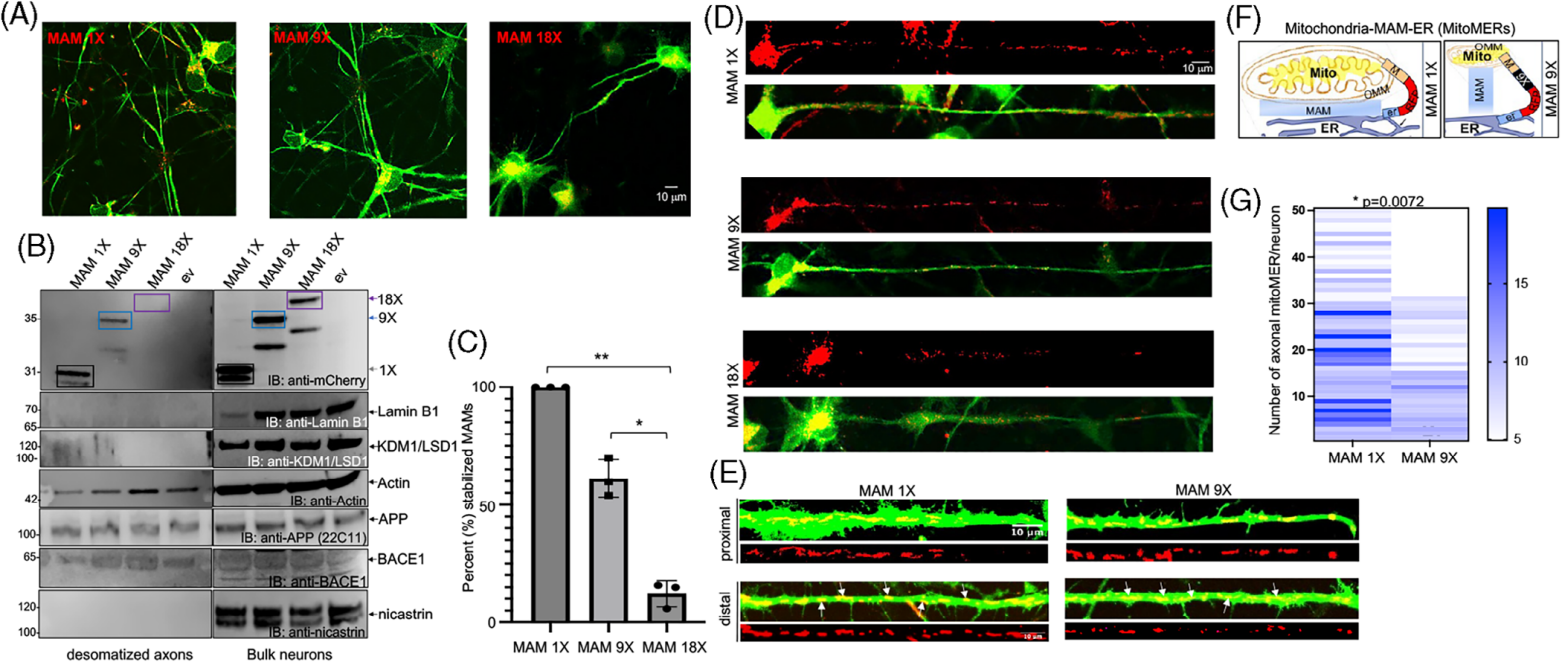
The stabilization of tight MAMs increased their axonal distribution compared to loose MAMs in ReN‐GA neurons. (A) Representative confocal images of 3D differentiated ReN‐GA neurons expressing MAM 1X, MAM 9X, or MAM 18X (red). ReN‐GA cells express GFP (green). (B) IB of protein extracts from bulk neurons and purified anucleated desomatized axons from 12‐day differentiated ReN‐GA cells expressing MAM 1X, MAM 9X, or MAM 18X probed with antibodies against mCherry (anti‐mCherry) that detected MAM 1X, 9X, and 18X‐tagged MAMs of three different gap widths. Dramatically increased levels of MAM 1X were detected in the desomatized axons compared to MAM 9X in the desomatized axons. The axonal levels of MAM 18X were severely reduced in desomatized axons. The nuclear membrane protein Lamin B1 (anti‐Lamin B1) or KDM1/LSD1 (anti‐KDM1/LSD1) were absent in the desomatized axons, confirming their purity. Actin levels remained equal. APP [anti‐APP (22C11)] and BACE1 (anti‐BACE1) levels remained equal in both desomatized axons and Bulk neurons from ReN‐GA expressing MAM 1X, 9X, or 18X. (C) Quantitation of the levels (%) of MAM 1X, MAM 9X, and MAM 18X in axons versus bulk neurons. *n* = 3, *, *p* < 0.05; **, *p* < 0.001. (D) Confocal images of single ReN‐GA neurons (green) expressing the RFP‐labeled stabilizers (red puncta). (E) Schematics of mitoMERs formed by MAM 1X or MAM 9X. (F) Representative axonal segments (∼100 µm) of proximal (close to the soma) or distal (close to the terminals) areas of axons. The green channel (GFP) was overexposed to show the axonal structures. The white arrows indicate the mitoMER organelles in the axons. The red puncta represent MAM 1X or MAM 9X‐labeled mitoMERs in axons. (G) Quantitative heatmap of the distribution of mitoMER organelles in axons labeled with MAM 1X or MAM 9X. Two‐way ANOVA. **, *p* < 0.001. Number of neurons (*n*) ≥ 5 from each experiment (triplicate experiments). The blue shading indicates the number of MAMs of a specific size per 100 µm axon. APP, amyloid precursor protein; GFP, green fluorescence protein; MAM, mitochondria‐associated ER membrane.

Our earlier report demonstrated that the upregulation of axonal MAMs via S1R dramatically increased, while downregulation severely reduced axonal Aβ levels.^9^ To examine whether the axonal MAM levels correlated with Aβ generation in the AD neurons, we determined the axonal distribution of the “*Aβ‐increasing*” tight MAMs and “*Aβ‐maintaining or ‐lowering*” basal or loose MAMs, respectively. Here, we measured the distribution of MAM 1X, MAM 9X, or MAM 18X‐labeled stabilized MAMs in the soma and axons. Confocal imaging revealed that although all three stabilizers were distributed in both soma and axons, their distribution in the axons varied (Figure 4A). Immunoblotting the desomatized axonal extracts with anti‐mCherry antibody detected both MAM 1X and MAM 9X in the axons albeit to a variable level (Figure 4B). In contrast, little or no MAM 18X was detected in the desomatized axons (Figure 4B). Quantitation revealed that 38.4 ± 6.3% of total MAM 1X and 22.6 ± 11% of total MAM 9X were detected in the desomatized axons compared to only 4.3 ± 2.7% (*p* = 0.003, *n* = 3) MAM 18X (Figure 4C). The 80%–90% loss of MAM 18X is consistent with several reports demonstrating that tight MAMs are formed between mitochondria and SER, the primary ER in axons, while the loose MAMs of gap widths 25–40 nm are formed between mitochondria and RER.^17^, ^18^, ^19^, ^20^ Detecting ∼23% of total MAMs of ∼25 nm gap widths in the axons is not surprising because several TEM studies have detected freely distributed SER and RER in the axons of cultured neurons, including in the axoplasm of desomatized axons of dorsal root ganglia neurons.^33^, ^61^, ^62^, ^63^ TEM images of desomatized axons from ReN‐GA neurons also detected both SER and RER, and MAMs formed between the ER and mitochondria with gap widths 6–30 nm (Figure S6).

To complement desomatization, we performed confocal microscopy of ReN‐GA‐derived neurons because confocal microscopy can distinguish between the somal and axonal compartments (Figure S7). We employed quantitative image analysis of the soma and axons, but not dendrites, because the relative MAMs in dendrites are significantly less abundant than those in soma or axons.^64^ Although high‐resolution microscopy, such as TEM, is commonly employed to assess the architectures of membrane contacts at the nanoscale level (Figures 1L and 2D), they cannot evaluate organelles' functional aspects and real‐time behavior within the cellular environment (*reviewed in* ^53^). Confocal microscopy offered a more accurate representation of MAMs than electron microscopy and provided insights into the role of mitochondria‐associated ER or plasma membrane‐associated ER membranes (MAMs or PAMs, respectively) in calcium homeostasis and lipid trafficking.^65^ While the somal distribution of the stabilizers was diffused, we detected discrete and quantifiable punctate labeling of MAM 1X and MAM 9X in the axons of 12‐day differentiated mature neurons (Figure 4D,E). MAM‐fused mitochondria‐ER, denoted here as MitoMERs, are expected to behave as single organelles (Figure 4F) in the axons. Quantitative image analysis revealed that 71.4 ± 10.3% of total MAM 1X‐labeled puncta and only 28.6 ± 8.2% of total MAM 9X‐labeled puncta were axonal (Figure 4G). We also detected significantly higher levels of large (> 5 µm2) MAM 1X‐labeled puncta compared to MAM 9X (Figure 4G, darker blue in the heatmap). In contrast, MAM 18X primarily labeled < 5 µm2 puncta in the axons (data not shown). The result is consistent with an earlier report in a cellular model of the peripheral immune cell (mast cell), RBL‐2H3, that controlling MAM's vertical thickness (gap width) also controls MAM's horizontal length. Precisely, RBL‐2H3 cells expressing MAM 1X stabilized ∼6 nm (vertical) tight MAMs that formed nearly five‐fold longer (horizontal length) MAMs compared to MAM 9X‐stabilized ∼25 nm gap width (1000 nm long vs. 221 ± 39 nm, respectively).^31^ Notably, mast cells are identified as potential therapeutic targets for AD.^66^ Stabilizing loose (∼40 nm) MAMs reduced their axonal distribution and ameliorated Aβ generation from pathogenic neurons. The finding is consistent with our previous report demonstrating a direct impact of axonal MAMs on axonal Aβ. We must highlight that the axons of ReN‐GA neurons do not form protective myelin sheaths in the absence of oligodendrocytes. Thus, the axonal Aβ accumulating in the axonal chambers in our microfluidic devices was likely the pool of Aβ formed inside axons or transported from the somatodendritic compartment being released into the axonal chambers. Extraneuronal Aβ is secondary to the generation of intraneuronal Aβ.^55^ Collectively, our results suggest that stabilizing tight MAMs increased the axonal distribution of the MitoMERs by approximately three‐fold compared to basal MAMs and exacerbated Aβ generation from pathogenic neurons. In contrast, the stabilization of loose MAMs severely impaired the axonal distribution of the loosely bound MitoMERs and Aβ levels in pathogenic neurons. Thus, the degree of MitoMER sizes or levels in the axons may be used as a metric to identify effective MAM modulators, such as S1R ligands, maintaining or lowering as opposed to increasing amyloid pathology.

### 3.5 Stabilization of MAMs significantly decreased the overall axonal transport rate of mitochondria, which was tightly associated with the ER as opposed to loosely associated with the ER or unbound to the ER

Several MAM modulators are under preclinical studies for cancer and metabolic disorders that target MAM structures by disrupting the ER‐mitochondria tethering proteins, the MAM‐resident proteins at their translational level, or the upstream signaling molecules, such as S1R.^25^ However, there is a lack of available functional assays to quantitatively measure the stabilization of MAMs due to their structural (gap width, length, or size) and functional (Aβ generation, Ca^2+^‐signaling, lipid transport) complexities. Moreover, the implications of the structural characteristics of the ER for MAM functions are not fully understood, possibly because the different conformations of the ER (rough vs. smooth) are not distinctly separated in most cells. However, neurons bypass this complication due to their intriguing cellular architecture characterized by pronounced polarization. The distinct separation of axons, predominantly housing SERs, from soma containing both SERs, RERs, and other forms of ER, such as cortical ER,^60^ provides an unprecedented opportunity to study the role of tight versus loose MAMs, which may have profound implications for mitochondrial integrity and transport in the development of neurodegenerative diseases.

**FIGURE 5:**
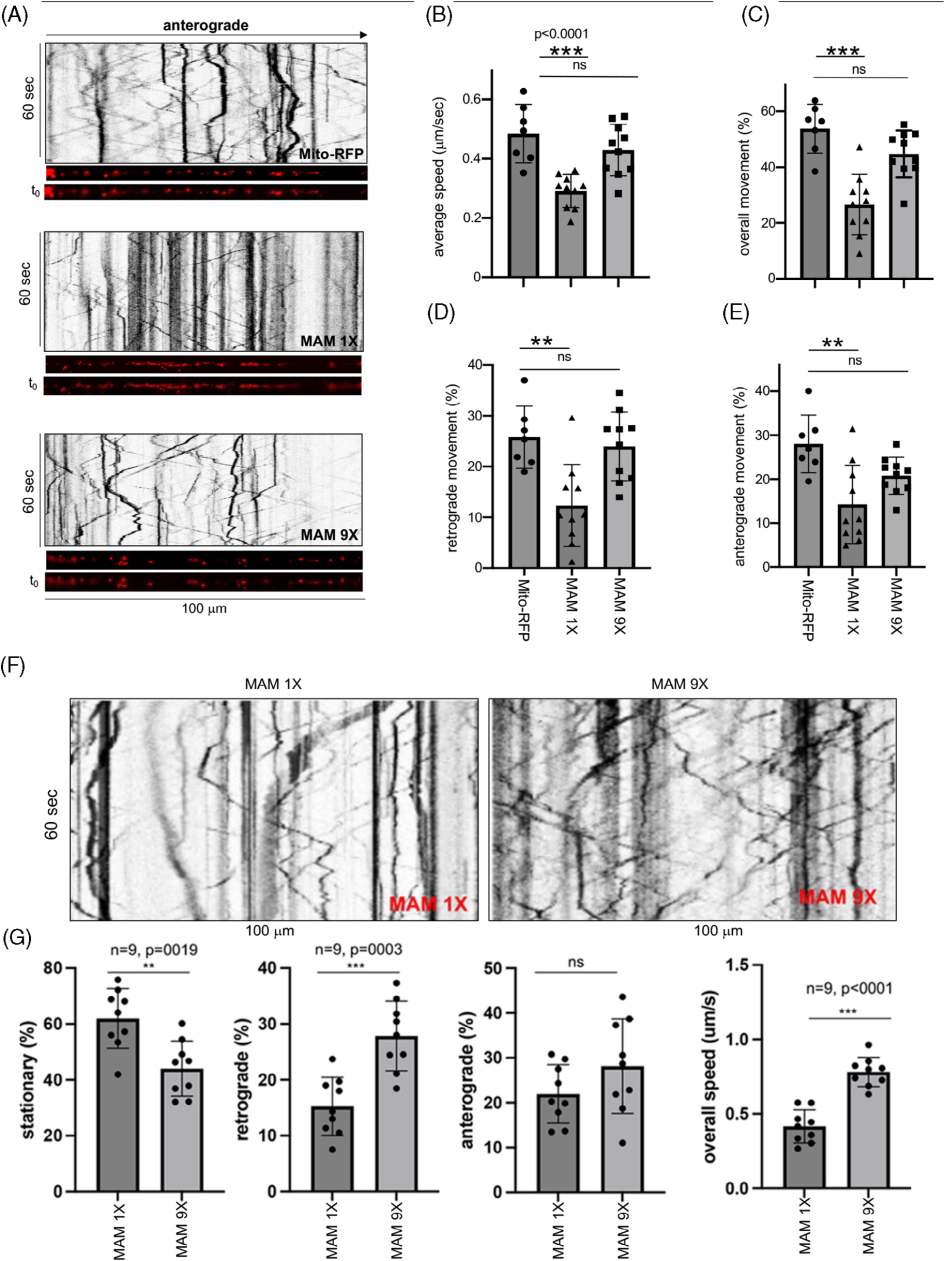
The stabilization of tight MAMs reduced their axonal mobility compared to free or loose MAMs in 3D‐differentiated ReN‐GA‐AD neurons. (A) Representative kymographs of axonal movement (stationary, retrograde, and anterograde) of Mito‐RFP, MAM 1X, and MAM 9X in axons of 12‐day differentiated ReN‐GA cells. ImageJ straightened live imaging videos (12 frames per second) and corresponding single‐frame images of the axons are presented under each kymograph. (B–E) Quantitative analysis of the overall speed (B) and percentage (%) of axonal movement (overall [C], retrograde [D], and anterograde [E]) of Mito‐RFP, MAM 1X and MAM 9X. (F, G) The kymographs (F) and the quantitative values (G) of the percent axonal movement and speed of the MitoMERs stabilized by MAM 1X or MAM 9X in 12‐day differentiated naïve ReN‐G neurons. The data are presented per 100 µm per 60 s. One‐way ANOVA. ***p < 0.0005, **p < 0.005. AD, Alzheimer's disease; ANOVA, analysis of variance; MAM, mitochondria‐associated ER membrane.

Here, we exploited the mitochondrial transport system in the axons (retrograde, anterograde, or both) that strongly impacts synaptic and neuronal function.^67^ Mitochondria transport along the microtubule assemblies via interaction with the adaptor proteins, Mitochondrial Rho (Miro) GTPases, and Milton (Miro/Milton) on one surface ^68^ while connecting to the ER via MAMs on the other surface. We assessed the impact of the ER‐fused pathogenic (“*Aβ‐increasing*”), basal (“*Aβ‐maintaining*”), and non‐pathogenic (“*Aβ‐lowering*”) MAMs on the mitochondrial mobility by performing a comprehensive quantitative analysis of the axonal mobility of the MitoMERs formed by tight, basal, or loose (∼6, ∼24, or ∼40 nm) MAMs. We employed live cell microscopy followed by kymography of Mito‐RFP, MAM 1X, MAM 9X, or MAM 18X‐labeled MitoMERs in the axons of 3D‐differentiated ReN‐GA cells to measure their overall movements in axons. We calculated the frequency of movement by dividing the number of moving or stationary organelles by the total number in the kymographs. The overall axonal velocity of the MAM 1X‐labeled MitoMERs was significantly lower approximately two‐fold compared to that of the Mito‐RFP‐labeled ER‐free mitochondria (Figure 5A,B, *p* < 0.0001) or MAM 9X‐labeled MitoMERs (Figure 5A,B, *p* = 0.032). The slightly reduced average axonal velocity of the MAM 9X‐labeled MitoMERs or MAM 18X‐labeled MitoMERs compared to Mito‐RFP‐labeled mitochondria was ns. (Figure 5A,B, ns). Quantitation of the percent overall, retrograde, and anterograde movement (Figure 5C–E, respectively) also reflected the results of the average axonal speed of the MitoMERs compared to free mitochondria (Table 1). These values can be used as a remarkable quantitative means to assess the degree of MAM stabilization that would increase or decrease Aβ in pathological conditions (Table 1). We also measured mitochondrial axonal transport rates upon stabilization of tight, basal, and loose MAMs in naïve ReN‐derived neurons (ReN‐G) that only expressed GFP (ReN‐G). We observed that the relative differences in axonal speeds (µm/s) between the ER‐free mitochondria (Mito‐RFP) or MitoMERs (MAM 1X, MAM 9X, or MAM 18X‐labeled) in ReN‐G neurons mirrored the transport patterns we observed in ReN‐GA neurons (Figure 5F and G; and Table 1). Given the consistent outcomes between naïve and APP_Swe/Lon_‐expressing ReN‐GA neurons, our results revealed that the ER‐connected mitochondrial axonal transport predominantly depends on MAM stabilization but not on APP_Swe/Lon_ or the resultant Aβ generation. Notably, the reported mitochondrial transport speed in cultured primary neurons were 0.6  ±  0.41 µm/s (anterograde) and 0.59  ±  0.35 µm/s (retrograde).^69^

**TABLE 1:** Percent axonal movement (overall, retrograde, anterograde) and average speed of Mito‐RFP and MitoMERs (light blue) stabilized by tight (MAM 1X) or loose (MAM 9X or 18X) MAMs in Ren GA (light blue) and naïve ReN in (dark blue).

Parameter	ReN GA					ReN GA (3D)	
	Overall (%)	Retrograde (%)	Anterograde (%)	Average speed (mm/s)	ReN (naïve)	Aβ_40_ (pM)	Aβ_42_ (pM)
Mito‐RFP	53.82 ± 3.3%	25.78 ± 2.31%	28.04 ± 2.48%	0.66 ± 0.03	0.69 ± 0.07	241.7 ± 26.74	13.77 ± 1.52
MAM 1X	26.6 ± 3.4% ^***^	12.33 ± 2.5% ^***^	14.27 ± 2.81% ^***^	0.3 ± 0.02^***^	0.43 ± 0.04^***^	377.2 ± 76.87^*^	26.62 ± 3.86^*^
MAM 9X	44.79 ± 2.6%^ns^	23.99 ± 2.17%^ns^	20.80 ± 1.33%^ns^	0.59 ± 0.02^ns^	0.62 ± 0.02 ns	158.8 ± 3.27^*^	17.01 ± 2.02^*^
MAM 18X	NA	NA	NA	NA	NA	61.93 ± 4.22^**^	3.33 ± 0.01^**^

*Note*: One‐way ANOVA. Total Aβ (Aβ_40_ and Aβ_42_) from 3D differentiated ReN GA neurons expressing the MAM stabilizers compared to neurons expressing Mito‐RFP. Paired *t*‐test.

Abbreviations: ANOVA, analysis of variance; MAM, mitochondria‐associated ER membrane; ns, not significant.

* *p* < 0.05.

** *p* < 0.001.

*** *p* < 0.0001.

## 4 DISCUSSION

**FIGURE 6:**
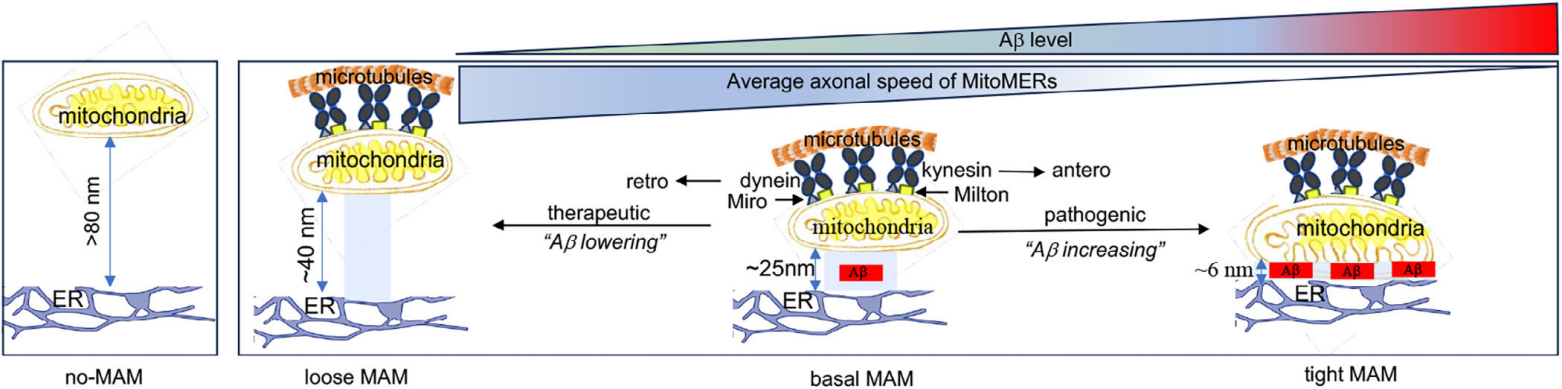
Schematic representation of MAM gap widths increasing or decreasing Aβ generation from AD neurons. Mitochondria and the ER separated by a distance > 80 nm do not form MAMs (no‐MAMs). The MAM gap width ranges from the tight (∼6 nm) to the loose (∼80 nm) with intermediates (10–80 nm). Most MAMs in the basal level are ∼25 nm thick (gap width). In the neurons expressing APP carrying FAD mutations, Aβ is generated in the MAMs at a basal level. “Tightening” the MAM gap width (vertical) increases the length (horizontal), resulting in an increased area between the ER and mitochondria to exacerbate the Aβ generation in the MAMs via yet unknown mechanisms. “Loosening” MAMs by increasing the vertical gap widths decreases the horizontal length and reduces Aβ generation. ER binding via MAMs counteracts the mitochondria's anterograde (antero) or retrograde (retro) drag on the microtubules exerted by myosin or kinesin, respectively, via the Miro/Milton complex in axons. The “tightening” of the MAMs increases the microtubule drag on mitochondria and lowers their axonal mobility. In contrast, “loosening” the MAMs restores mitochondria's axonal mobility. An effective modulator that may regulate MAM stabilization by “loosening” but not disrupting MAMs may switch “Aβ‐*generating*” pathogenic MAMs to “Aβ‐*lowering*” therapeutic MAMs by crossing the threshold of Aβ “*maintaining”* or “*neutral*” MAMs. The dose and effectiveness of the “Aβ‐lowering” MAM modulators can be determined by measuring the axonal mobility of the ER‐fused mitochondria (MitoMERs) after treating our 3D neural model of AD with potential MAM modulators. 3D, three‐dimensional; AD, Alzheimer's disease; APP, amyloid precursor protein; ER, endoplasmic reticulum; FAD, familial AD; MAM, Mitochondria‐associated ER membrane.

“MAM hypothesis” proposes that AD MAMs play a critical role in Aβ generation to initiate the pathogenic cascade of AD.^1^, ^4^ Using inducible MAM stabilizers composed of rapamycin (Rapa) and rapalog (Log)‐inducible FRB and FKBP dimerization components in N2A_APP_ cells we have shown that Rapa/Log treatment tightens ER‐mitochondria contacts, enhancing Aβ_40_ generation in a dose‐dependent manner (Figures 1, 2, 3). This observation provides the first proof of concept that tightening the MAM gap width contributes to amyloidogenic processes. Pharmacological anti‐dementia agent rivastigmine that modulates MAMs by perturbing the MAM‐anchoring MFN2 and MAM‐resident sterol O‐acyltransferase 1 (SOAT1),^49^ lowered Aβ generation from a 3D ReN‐GA neuronal model of AD (Figure 1). Further, studies using fixed biological linkers to stabilize MAMs with specific gap widths (either tight ∼6 nm or loose ∼40 nm) in the 3D ReN‐GA neuronal model of AD demonstrated that tight MAMs significantly exacerbated, while loose MAMs ameliorated Aβ (Aβ_40_ and Aβ_42_) production (Figure 3). Our study has demonstrated that the stabilization of the “*Aβ‐increasing*” tight MAMs increased and the “*Aβ‐lowering*” loose MAMs decreased their axonal distribution compared to basal MAMs (Figure 4). Moreover, the stabilization of tight MAMs significantly reduced mitochondrial mobility in axons compared to free mitochondria or mitochondria bound to the ER via basal (∼25 nm) MAMs (Figure 5). The axonal mobility of mitochondria fused to MAMs by MAM 18X (gap width ∼40 nm), on the other hand, exhibited axonal mobility comparable to both basal MAM‐fused and ER‐free mitochondria (data not shown). Together, our findings indicate that the “loosening” or “tightening” of axonal MAMs impacts Aβ generation and mitochondrial mobility in AD neurons (Figure 6), which is consistent with our previous report demonstrating that the inactivation of S1R downregulated axonal MAM assembly and resulted in a severe reduction in axonal Aβ levels.^9^

The modulation of MAMs is an emerging area of research with potential implications for various disorders, including cancer, metabolic disorders, and neurodegenerative diseases.^25^ In a recent study, Carpio et al. reported that MAMs that form between tightly apposed (∼10 nm) ER‐mitochondria, denoted as “full MAMs,” are apoptotic. In comparison, MAMs that form between loosely apposed (∼25 nm) ER and mitochondria contacts, denoted “defective” or “medium” MAMs, are antiapoptotic.^23^ Modulating MAM stabilization, rather than disrupting it, could be a valuable therapeutic strategy for AD, as destabilizing MAMs might interfere with important cellular processes necessary for cell survival.^19^ As a proof‐of‐concept, we have demonstrated that the pharmacological agent rivastigmine, known to impact MAMs by regulating MAM‐anchoring MFN2 and MAM‐resident SOAT1/ACAT1,^49^ lowered Aβ generation from our 3D ReN‐GA neurons (Figure 1). Notably, pharmaceutical inhibitors of the ACAT1 (the product of *SOAT1* gene), such as CP‐113,818, CI‐1011, K‐604, or F12511 reduced amyloid pathology by lowering neuronal Aβ generation or by enhancing Aβ clearance through upregulated microglial MAMs.^70^, ^71^, ^72^ Given the involvement of both neuronal generation and microglia clearance of Aβ, further exploration of the MAM axis in AD could lead to more effective therapeutic interventions.

Impaired mitochondrial axonal transport is an early event in AD, preceding toxic protein accumulation and synaptic dysfunction, and is strongly associated with increased Aβ, phospho‐Tau levels, and oxidative stress.^73^, ^74^, ^75^, ^76^ Primary neuron studies from AD models, such as Tg2576 mice, show decreased anterograde mitochondrial movement and defective mitochondrial function,^74^ contributing to the loss of axonal integrity. Microtubule tracks essential for mitochondrial transport are also disrupted in AD. We hypothesize that the increased bulkiness of ER‐mitochondria connected by tight MAMs in axons may counteract the microtubule‐driven transport of mitochondria, slowing their mobility. Indeed, the stabilization of tight MAMs significantly reduced the axonal velocity of mitochondria compared to those free from ER connections (Figure 5). These findings suggest that the long contact sites between the tightly apposed ER and mitochondria upon stabilization with MAM 1X may result in the detachment of mitochondria from the microtubules, which would switch mitochondria from a mobile to a stationary state. Recent electron microscopy studies have identified extensive ER wrapping around mitochondria in hepatocytes, known as wrappers.^77^ While wrappER has not been observed in neurons, we found ER structures wrapped around mitochondria in ReN‐GA neurons (Figure S4B, white arrowheads). A more comprehensive investigation of wrappER in neurons could expand our understanding of MAMs' role in mitochondrial dynamics and amyloidogenesis, potentially offering novel therapeutic approaches for AD and other neurodegenerative diseases.

Pharmacological agents like the dynein‐antagonist LDC‐3/Dynarrestin, metformin, and sulforaphane, which modulate ER‐mitochondria contacts by regulating MAM‐tethering proteins, are currently undergoing preclinical and clinical trials for cancer and metabolic disorders.^26^, ^78^, ^79^, ^80^, ^81^, ^82^, ^83^ Despite the availability of various MAM modulators, few attempts have been made to test their ability to destabilize MAMs and reduce AD pathology. This is likely due to the structural complexity of MAMs, which makes drug targeting challenging. However, the development of a structural systems pharmacology approach, which considers the specific properties of drug targets and their cellular environments,^84^, ^85^ may help overcome these obstacles. Currently, no reliable real‐time quantitative assays exist for measuring MAM stabilization. While traditional techniques like electron and super‐resolution microscopy detect MAMs at the nanoscale, they lack quantitative accuracy. Our study introduces live‐cell imaging and kymography‐based quantification of axonal mitochondrial velocity as a novel method for assessing MAM stabilization. Table 1 provides detailed axonal velocities linked to varying MAM associations. This can be used as a remarkable quantitative method to assess the optimal MAM stabilization needed to cross the threshold from pathogenic (Aβ *increasing*) to nonpathogenic (Aβ *maintaining* or Aβ *lowering*) MAMs.

The functional diversity of MAM structures, specifically their length and gap width, has been identified as a regulator of critical cellular processes, including calcium signaling between the ER and mitochondria and apoptosis.^86^ Mitochondrial calcium (Ca^2+^) dysregulation is an early marker of AD.^87^ The “calcium hypothesis” of AD suggests that aberrant calcium signaling contributes to disease progression.^88^ Previous research from Hajnóczky lab reported that the tightening MAM gap widths by MAM 1X did not significantly affect the resting or agonist‐stimulated cytosolic [Ca^2+^]_c_ levels in cultured cells,^20^ implying that MAM gap width modulation alone may not directly influence overall Ca^2+^ homeostasis. However, a recent study on astrocytes and neurons from mouse brains expressing the AAV OMM‐RFP‐ER (AAV‐MAM 1X) linker found that stabilizing tight MAMs resulted in a modest [Ca^2+^]_c_ change,^89^ though it had limitations in accurately measuring [Ca^2+^] levels. Future studies using advanced imaging techniques such as FLIM are needed to better assess the role of local Ca^2+^ dynamics at ER‐mitochondria contact sites. Although the contact areas represent only a small fraction of the total mitochondrial surface,^31^, ^90^ further studies are needed to assess the interplay between local Ca^2+^ dynamics and MAM structures.

In addition to calcium regulation, the role of protein palmitoylation in MAM stabilization and Aβ production is another critical area of interest. Palmitoylation is a reversible post‐translation modification that impacts several neurodegenerative diseases, including AD, Parkinson's disease (PD), and Huntington's disease (HD) (reviewed in refs. [^91^, ^92^]). We and others have reported that APP and the enzymes responsible for its amyloidogenic processing, the β‐ (BACE1) and γ‐secretase components (nicastrin and APH‐1), are palmitoylated, which is important for their association with the cholesterol‐rich LRs or Aβ generation in vitro and in vivo.^35^, ^36^, ^93^, ^94^, ^95^ APP undergoes lumenal palmitoylation in the ER.^35^ Upregulation of MAMs increased the half‐life of palmitoylated APP (palAPP), but not total APP.^9^ BACE1 and components of γ‐secretases are all found enriched in MAMs.^96^ An in‐depth understanding of the processes that modulate MAM structures and regulate BACE1‐ or γ‐secretase‐mediated processing of APP or palAPP for Aβ generation, specifically in the axons, could significantly impact the development of early therapies for AD. Several proteins, such as calnexin, and thioredoxin‐related transmembrane protein (TMX1) also undergo lumenal palmitoylation and are targeted to the MAMs in a palmitoylation‐dependent manner.^97^ Palmitoylated calnexin binds to the MAM‐resident sarco/ER Ca^2+^ ATPase2b (SERCA2b) and promotes Ca^2+^ flux toward the ER.^98^ Palmitoylation of TMX1, on the other hand, increases its affinity for SERCA2b and re‐directs Ca^2+^ flux into mitochondria.^99^ Hence, tight control of calnexin and TMX1 palmitoylation and MAM localization appears paramount for restoring Ca^2+^ homeostasis in AD, possibly at the early stages.

The role of axonal Aβ in AD is unclear. Some studies suggest that APP, BACE1, and γ‐secretase are transported together along axons, allowing for Aβ production during axonal transport.^100^, ^101^ However, a later study has challenged this idea, by failing to detect the co‐transportation of either PS‐1 or BACE1 with APP in axons.^102^ Further investigation is needed to resolve these conflicting findings and clarify the role of MAMs in axonal Aβ production in AD neurons.

In conclusion, the modulation of MAM structures, specifically through tightening or loosening MAM gap widths, has a profound impact on both Aβ generation and mitochondrial mobility in neurons. Pharmacological interventions targeting MAM stabilization, rather than complete disruption, offer promising therapeutic potential for AD. Future research into MAM dynamics, particularly in axonal transport and calcium regulation, could provide new insights into the development of early diagnostic tools and treatments for AD.

## CONFLICT OF INTEREST STATEMENT

All authors report no conflict of interest. Author disclosures are available in the Supporting Information.

## Supporting information



Supporting Information

Supporting Information

Supporting Information

## Data Availability

The datasets generated during the current study are available from the corresponding author upon request. The expression plasmids of the constitutive or inducible MAM stabilizers are available from G.H. or the corresponding author upon request. Custom macros prepared for ImageJ to measure axonal mobility and the codes will be available from the published method.^103^
