# Supplementary material for: Stabilization of mitochondria‐associated endoplasmic reticulum membranes regulates Aβ generation in a three‐dimensional neural model of Alzheimer's disease
Source: Alzheimers Dement. 2024 Dec 23;21(2):e14417. doi: 10.1002/alz.14417 (PMC11848173; doi:10.1002/alz.14417)
Supplement: Supplementary file 2 — Supporting Information [file ALZ-21-e14417-s002.pptx]

## Slide 1
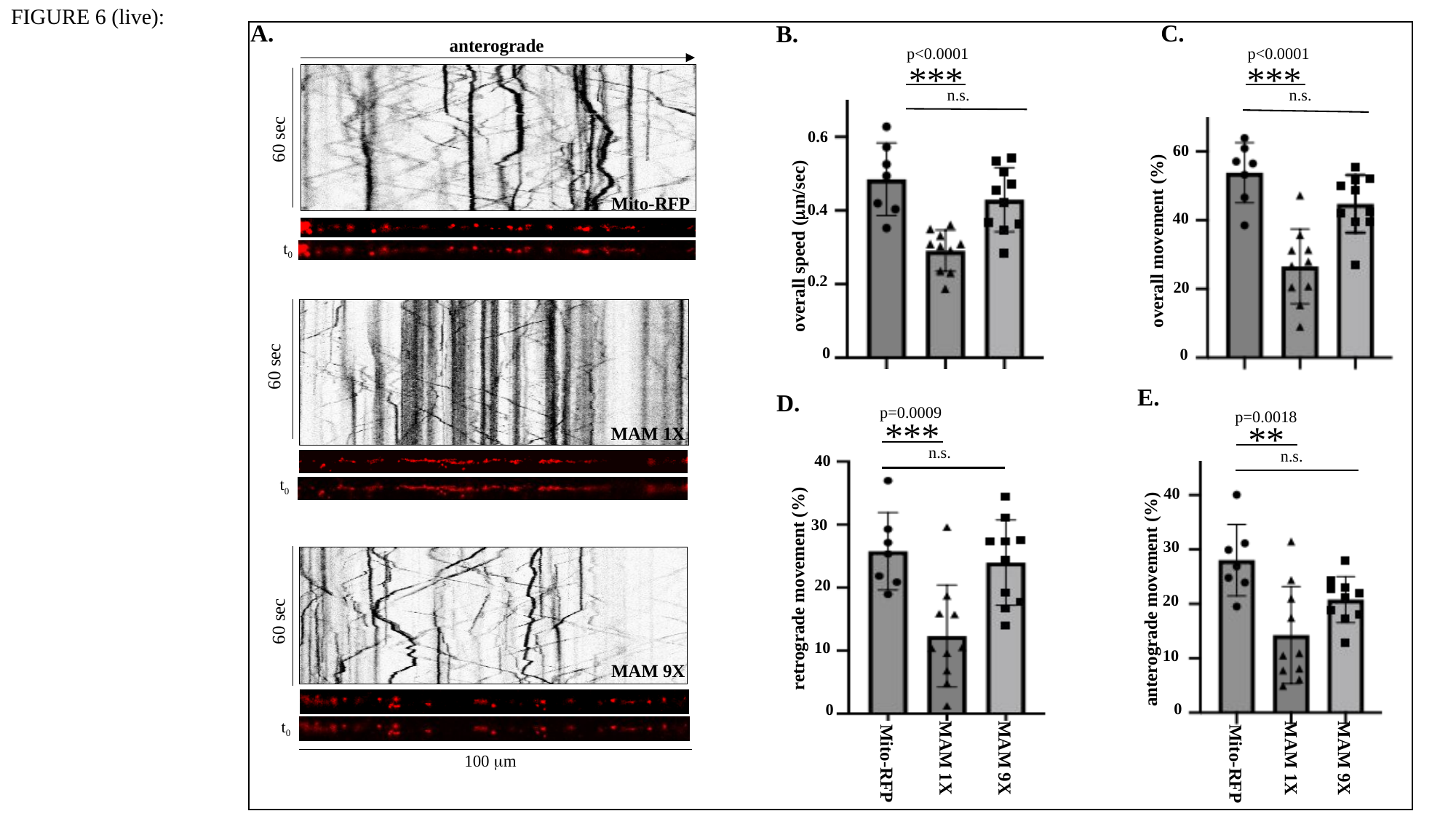

FIGURE 6 (live):
A.
C.
B.
anterograde
p<0.0001
p<0.0001
***
***
n.s.
n.s.
60 sec
0.6
60
Mito-RFP
0.4
40
overall movement (%)
overall speed (mm/sec)
t0
0.2
20
0
0
60 sec
E.
D.
p=0.0009
p=0.0018
***
**
MAM 1X
n.s.
n.s.
40
t0
40
30
30
20
retrograde movement (%)
anterograde movement (%)
20
60 sec
10
10
MAM 9X
0
0
t0
MAM 1X
MAM 9X
MAM 1X
MAM 9X
100 mm
Mito-RFP
Mito-RFP

## Slide 2
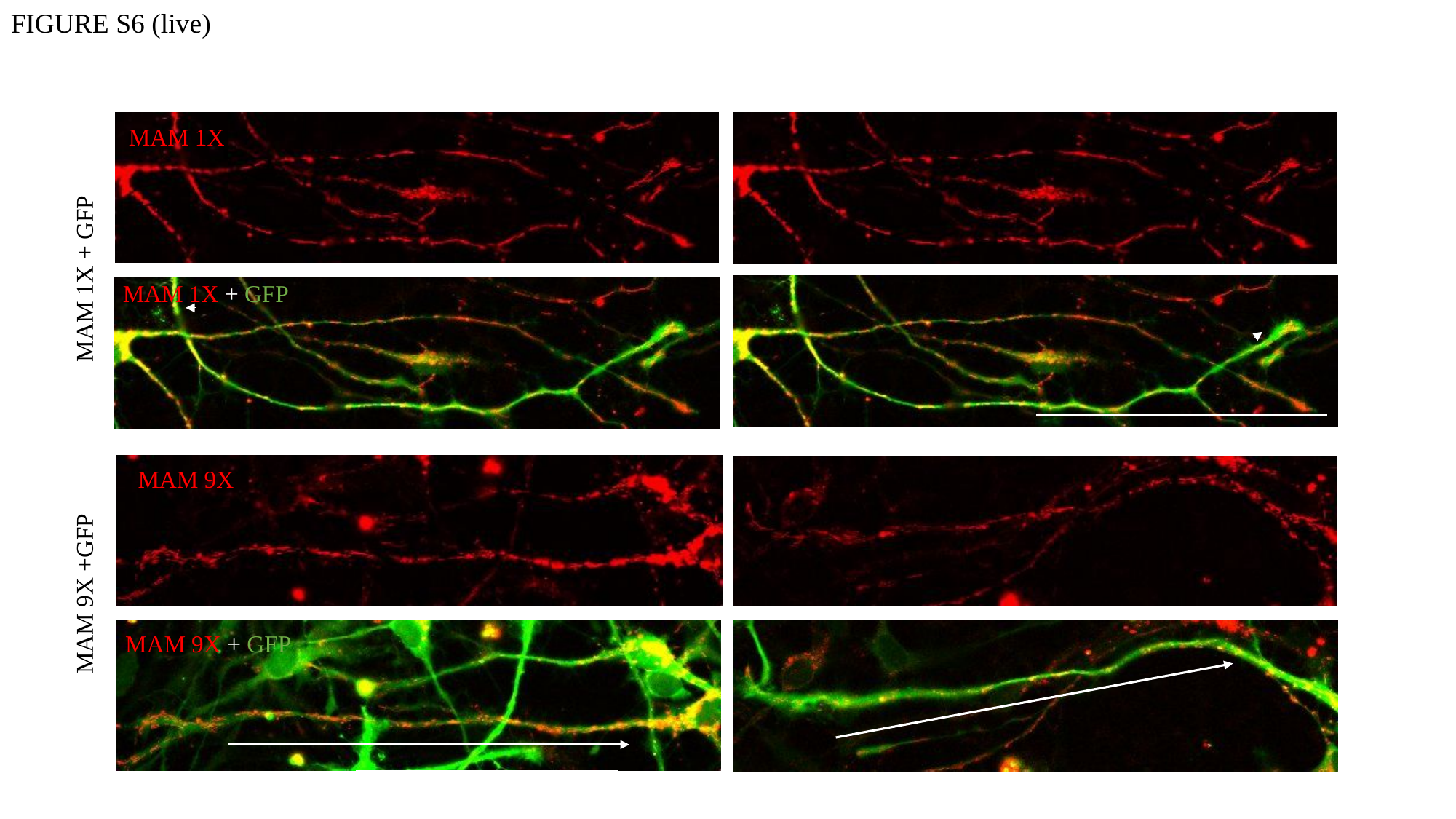

FIGURE S6 (live)
MAM 1X
MAM 1X + GFP
MAM 1X + GFP
MAM 9X
MAM 9X +GFP
MAM 9X + GFP
